# Scalable and Healable Gradient Textiles for Multi-Scenario Radiative Cooling via Bicomponent Blow Spinning

**DOI:** 10.1007/s40820-025-01947-2

**Published:** 2025-12-05

**Authors:** Baiyu Ji, Yufeng Wang, Ying Liu, Yongxu Zhao, Fankun Xu, Jian Huang, Yue-E. Miao, Chao Zhang, Tianxi Liu

**Affiliations:** 1 State Key Laboratory of Advanced Fiber Materials, College of Materials Science and Engineering, Donghua University, Shanghai, 201620 People’s Republic of China; 2 Key Laboratory of Synthetic and Biological Colloids, Ministry of Education, School of Chemical and Material Engineering, Jiangnan University, Wuxi, 214122 People’s Republic of China; 3 Department of Materials Science and Engineering, City University of Hong Kong, 83 Tat Chee Avenue, Hong Kong, People’s Republic of China

**Keywords:** Gradient cooling textile, Bicomponent blow spinning, Janus spectral selectivity, Radiative heat exchange, Multi-scenario radiative cooling

## Abstract

Radiative cooling textiles with spectrally selective surfaces offer a promising energy-efficient approach for sub-ambient cooling of outdoor objects and individuals. However, the spectrally selective mid-infrared emission of these textiles significantly hinders their efficient radiative heat exchange with self-heated objects, thereby posing a significant challenge to their versatile cooling applicability. Herein, we present a bicomponent blow spinning strategy for the production of scalable, ultra-flexible, and healable textiles featuring a tailored dual gradient in both chemical composition and fiber diameter. The gradient in the fiber diameter of this textile introduces a hierarchically porous structure across the sunlight incident area, thereby achieving a competitive solar reflectivity of 98.7% on its outer surface. Additionally, the gradient in the chemical composition of this textile contributes to the formation of Janus infrared-absorbing surfaces: The outer surface demonstrates a high mid-infrared emission, whereas the inner surface shows a broad infrared absorptivity, facilitating radiative heat exchange with underlying self-heated objects. Consequently, this textile demonstrates multi-scenario radiative cooling capabilities, enabling versatile outdoor cooling for unheated objects by 7.8 °C and self-heated objects by 13.6 °C, compared to commercial sunshade fabrics.

**Supplementary Information:**

The online version contains supplementary material available at 10.1007/s40820-025-01947-2.

## Highlights

- An ultra-flexible and gradient-structured textile is fabricated through bicomponent blow spinning, enabling the scalable production and in situ healing of the textile.
- The gradient in fiber diameter of this textile creates a hierarchically porous structure in the region exposed to sunlight, resulting in a solar reflectivity of 98.7% on its outer surface.
- The gradient in the chemical composition of this textile exhibits asymmetric spectral selectivity, wherein the outer surface offers high mid-infrared emissivity while the inner surface enables efficient radiative heat exchange.
- The gradient textile demonstrates multi-scenario radiative cooling capabilities, enabling simultaneous cooling for unheated and self-heated outdoor objects.

**Supplementary Information:**

The online version contains supplementary material available at 10.1007/s40820-025-01947-2.

## Graphical abstract

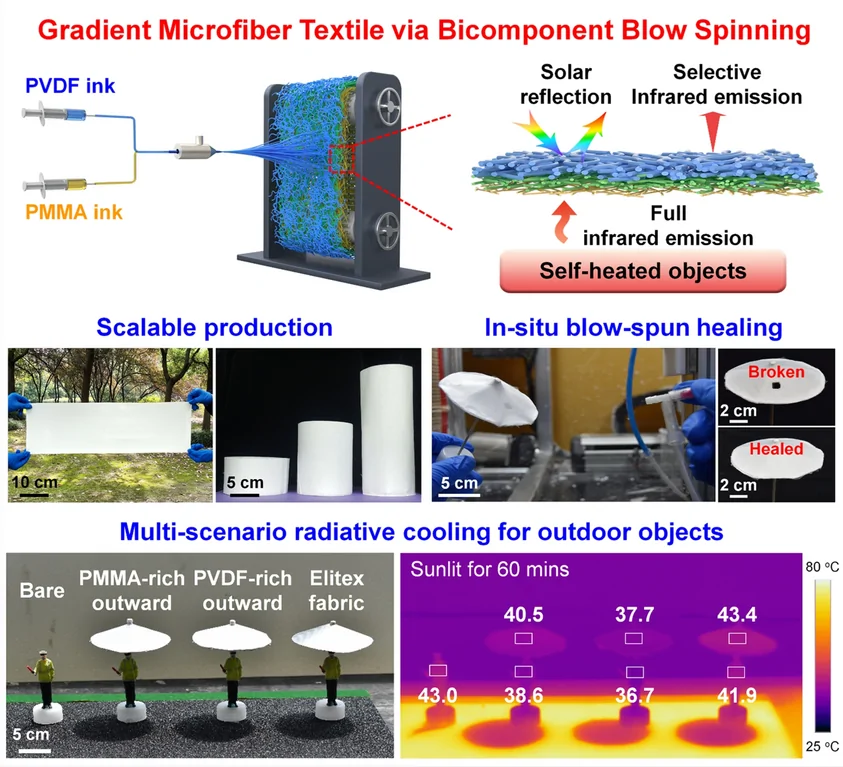

## Introduction

Global warming and extreme climate are increasingly prevalent phenomena, posing risks to the well-being and comfort of individuals and devices exposed to prolonged intense sunlight and high temperatures [1, 2]. This issue not only threatens personal health but also jeopardizes the reliable operation of outdoor electronics and equipment over extended periods [3, 4]. It is crucial to acknowledge the substantial cooling differences between outdoor and indoor environments. Traditional active cooling methods like sprinkler systems or air conditioning units are neither cost-effective nor practical for achieving efficient cooling in outdoor settings [5, 6]. Consequently, it is desirable to develop technologies with low or no energy consumption for cooling heat-generating objects outdoors [7]. Passive daytime radiative cooling stands out as a promising solution, leveraging the manipulation of reflectance under solar exposure and emissivity within the atmospheric transparent window to achieve energy-efficient cooling without the need for external power sources [6–11]. This approach addresses concerns related to energy efficiency, environmental impact, and safety during extreme summer heat conditions. Among the various radiative cooling materials, efficient Mie scattering is achieved by incorporating nano/micrometer pore structures within bulk materials, enhancing their sunlight reflectivity [12–20]. However, research indicates that the optimization of multi-level pore structures within porous structural materials has already reached the upper limit for enhancing their solar reflective capabilities [15–18]. Moreover, outdoor cooling applications frequently encounter diverse cooling requirements across various scenarios, necessitating materials that offer effective radiative thermal management for objects that are not only externally heated but also self-heated [21, 22].

Textiles are defined as film-like or felt-like structures comprising fibers that are bonded or combined through physical or chemical methods [23–28]. The arrangement of fibers within these textiles, whether regular or random, generates diverse pore structures, leading to textiles with high porosity and significant gas–solid interfaces [29–31]. The morphology of the fiber structure and the type of fiber arrangement in the textile can be readily adjusted to align the fiber aperture and pore distribution with the solar spectrum for efficient Mie scattering. Additionally, textiles with tailored chemical compositions and high-surface-area porous structures are anticipated to enhance their emissivity in the atmospheric window, meeting the essential criteria for radiative cooling [31–33]. Notably, textiles are characterized by their flexibility, lightweight nature, and thermal insulation characteristics, making them an ideal material for effective outdoor radiative thermal management [34–36]. However, traditional methods of fabricating textiles often result in a narrow distribution of fiber diameters to achieve a uniform weaving structure, limiting their ability to comprehensively reflect broadband sunlight [24, 30, 37]. Furthermore, conventional textiles designed for radiative cooling, focusing on the outdoor-facing side with selective high emission in the atmospheric window, struggle to effectively cool self-heated objects like electronic devices and human bodies [38, 39]. Spectrally selective radiative cooling materials frequently fail to release internal heat promptly, posing risks to effectively cooling both individuals and equipment. Hence, the development of Janus emissive properties on both sides of radiative coolers is crucial to facilitate internal heat dissipation. These Janus structures efficiently absorb internal thermal radiation and radiate excess heat to the cold outer space, thereby alleviating indoor heat accumulation [22, 40–42]. Although multilayer structures offer the ability to modulate internal and external spectral properties, techniques like magnetron sputtering and chemical deposition, which are commonly used in preparation, are costly and intricate [39]. Similarly, the production of asymmetric radiatively cooling emitters through nonwoven methods like electrospinning encounters difficulties in both preparation and structural modulation [22, 39–41]. Moreover, simple multilayer structures struggle to provide sufficient scattering units to maximize sunlight scattering efficiency [37, 42]. Therefore, achieving cost-effective and large-scale fabrication of textiles with precisely tailored asymmetric spectral selectivity and resolving the conflict between the requirement for complete solar reflection and the radiative heat exchange with internal self-heated objects poses a significant challenge.

Herein, an ultra-flexible and large-area gradient microfiber textile (GMFT) was produced through a bicomponent blow spinning strategy, which involves the real-time blending of two distinct inks through a specially designed bicomponent feeding and mixing system. This fabrication method enables the creation of the microfiber textile with precisely tailored dual gradients in chemical composition and fiber diameter, along with post-healing capabilities. The GMFT displays enhanced solar reflection on its outer surface due to the formation of the gradient in fiber diameter along the sunlight incident pathway, demonstrating high solar reflectivity (98.7%) and high mid-infrared emission (95.3%). In addition, the GMFT shows Janus spectral characteristics on the outer and inner surfaces, namely the outer surface facilitates efficient radiative cooling while the inner surface exhibits complete mid-infrared absorption for achieving efficient radiative heat exchange with underlying self-heated objects. As a result, the obtained GMFT demonstrates outdoor multi-scenario radiative cooling capabilities for both unheated and self-heated objects.

## Experimental Section

### Materials

Poly(vinylidene fluoride) (PVDF, KYNAR 762) was obtained from Arkema (France). Polymethyl methacrylate (PMMA, BA141, *Mw* = 100,000 g mol^−1^) was obtained from LG Chem (Korea). DMF (AR, ≥ 99.5%) was provided by Sinopharm Chemical Reagent Co., Ltd. (China). MA (GC, ≥ 99%) was provided by Aladdin Reagent Co., Ltd. (China). Elitex fabrics (EB 5448ALU-1% O/F; composition: 76% polyvinyl chloride, 22% polyester (± 5%), and 2% aluminum coating; thickness: 0.66 mm (± 5%); openness factor: ~ 1%) were provided by Elitex Co., Ltd. (China).

### Preparation of the GMFT

The spinning inks were prepared as follows: PVDF was dissolved in a DMF/MA mixed solvent with a volume ratio of 2:3, while PMMA was dissolved in a DMF/MA mixed solvent with a volume ratio of 3:2. These solutions were individually loaded into 20 mL syringes for thorough mixing and blow spinning using a bicomponent injection system. The spinning chamber maintained a relative humidity of 40%, a temperature of 30 °C, and a gas pressure between 0.1 and 0.2 MPa. To fabricate the GMFT, the spinning injection pump of PVDF ink was initially set to 30 mL h^−1^ for 10 min, followed by a gradual decrease at a rate of 0.5 mL min^−1^ until reaching 0 mL h^−1^. Simultaneously, the spinning injection pump of PMMA ink started at 0 mL h^−1^ and increased to 30 mL h^−1^ at a similar decreasing rate of 0.5 mL min^−1^, maintaining this speed for an additional 10 min.

### Preparation of the MFT_F_ and MFT_A_

Pure PVDF microfiber textiles (MFT_F_) were produced by blow spinning of the PVDF ink of varying concentrations, denoted as MFT_F10_, MFT_F15_, MFT_F20_, and MFT_F25_ to represent the PVDF weight fraction in the PVDF ink (10 wt%, 15 wt%, 20 wt%, and 25 wt%). Similarly, pure PMMA microfiber textiles (MFT_A_) were generated using PMMA ink at different concentrations, labeled as MFT_A10_, MFT_A15_, MFT_A20_, and MFT_A25_ based on the PMMA weight fraction in the PMMA ink. Comparative samples, designated as MFTF_7A3_, MFT_F5A5_, and MFT_F3A7_, were produced by blow spinning of the mixed solution with a total solid content of 15 wt%, featuring PVDF/PMMA weight ratios of 7:3, 5:5, and 3:7, respectively. The spinning chamber was regulated to uphold a relative humidity of 40%, a temperature of 30 °C, and a gas pressure of 0.15 MPa. The spinning injection pump for various spinning inks was set at a rate of 30 mL h^−1^. All textiles were removed from the nylon mesh of the collection device and placed in an oven at 50 °C to eliminate any residual solvent.

### Materials Characterization

The morphologies of the surface and cross section of the GMFT were characterized using a scanning electron microscope (SEM, JSM-7500F). The fiber-diameter distribution was statistically analyzed using Nano Measurer software. Tensile properties were evaluated with a universal testing machine (CMT 4204, SUNS) at a strain rate of 5 mm min^−1^ at 25 °C. The water contact angles of GMFT were measured using a contact angle goniometer (OCA40 Micro). Infrared images of the GMFT were captured with an infrared thermal camera (FOTRIC 220S). The pore size distribution of GMFT was determined using a capillary flow porosimeter (PMI, CFP-1500AEX). The viscosity of the spinning solution was measured with a rheometer (Anton Paar, MCR 302) equipped with a 25 mm diameter parallel plate. The glassy transition temperature was assessed using a differential scanning calorimeter (DSC 4000) at a heating/cooling rate of 5 °C, within the range of − 70 to 160 °C. Solar reflectance spectra of GMFT were obtained using a UV–Vis–NIR spectrophotometer (UV3600) equipped with a standard white plate (Diffuse Lambertian PTFE) over a wavelength range of 300–2500 nm. The refractive indices and extinction coefficients of PMMA and PVDF were measured by ellipsometry (J. A. Woollam IR-VASE). The infrared emission spectra of GMFT were recorded using a Fourier-transform infrared spectrometer (VERTEX80v) with a gold integrating sphere in the range of 2.5–20 μm. According to Kirchhoff's law, emissivity at thermal equilibrium is calculated as ε(A) = 1-R-T, where ε(A) is absorbance, R is reflectance, and T is transmittance. Note that the absorbance and emissivity of an object concerning light are equivalent. Tests were conducted by varying the placement angle of the GMFT to obtain infrared emission spectra at different angles. Thermal conductivity measurements of GMFT, Elitex fabrics, MFT_A15_, and MFT_F15_ were performed using a hot disk thermal analyzer (Hot Disk TPS 2500S) employing the transient plane source method.

## Results and Discussion

### Fabrication and Characterization of the GMFT

**Fig. 1:**
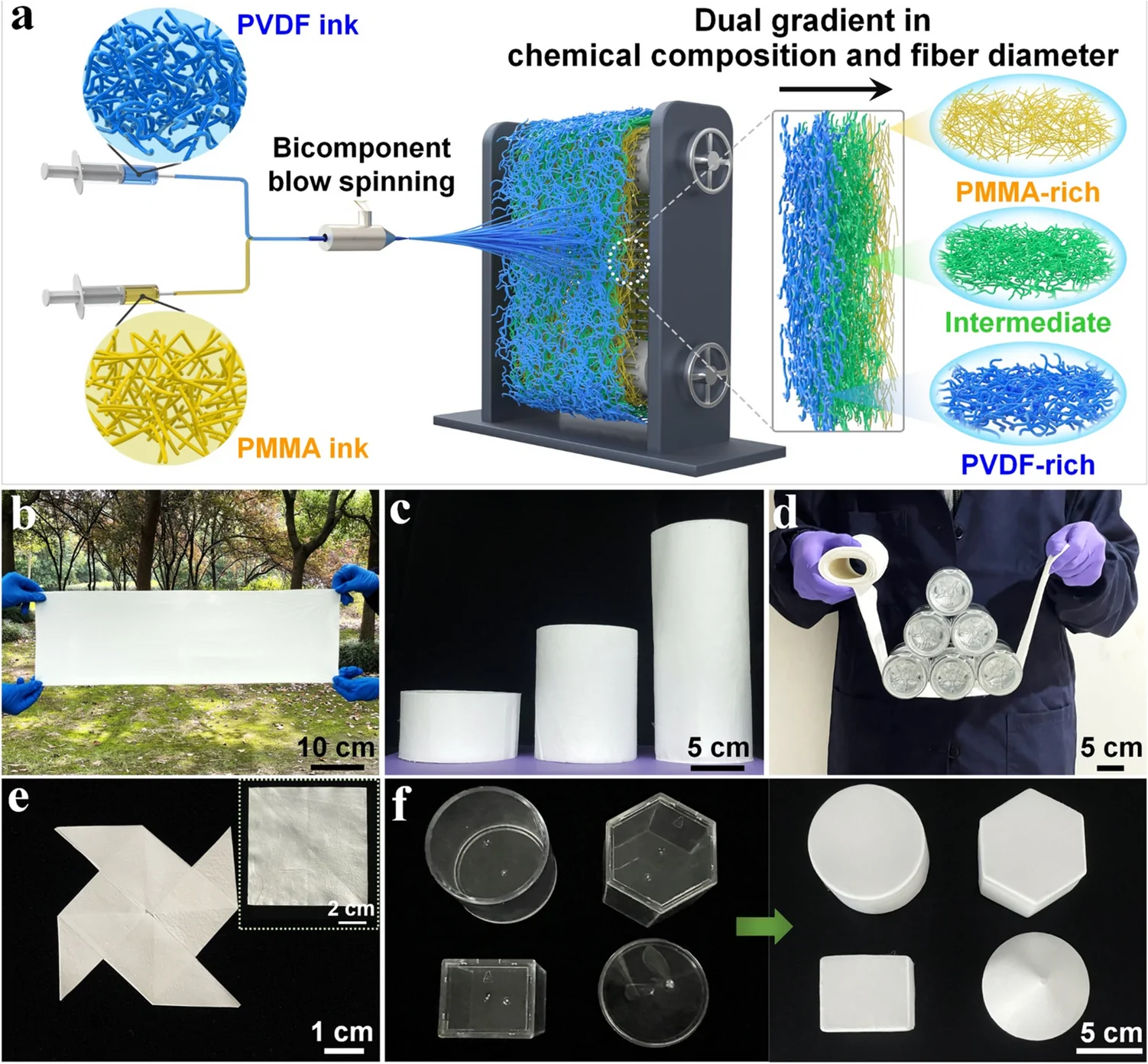
Preparation and characterization of GMFT. **a** Schematic diagram illustrating the bicomponent blow spinning process. Photographs of the GMFT: **b** in large sizes, **c** rolled up bundles, **d** supporting a 5 kg load. **e** Photographs of the GMFT demonstrating folding. Inset displaying the opened GMFT after folding. **f** Photograph depicting the fabrication of GMFT on surfaces of various shapes

Figure 1a depicts the schematic of the bicomponent blow spinning process for fabricating the dual-gradient microfiber textile (GMFT). Initially, two separate polymer spinning solutions were real-time and thoroughly mixed by controlling their respective injection rates. Microfibers with continuously varying diameters were successfully produced through the continuous blow spinning of the real-time mixed spinning solution, leading to the establishment of a gradient distribution of chemical composition and fiber diameter across the thickness of the resulting textiles. Among the GMFT, the blue color denotes the PVDF-rich region, the yellow color represents the PMMA-rich region, and the green color signifies the microfibers containing both components. Enlarged images highlight the gradient changes in fiber diameter. Scanning electron microscope (SEM) images of the different regions in the cross section of the GMFT illustrated the gradient variations in fiber diameter (Fig. S1). In Fig. 1b, an optical image shows the fabricated GMFT in large sizes of 80 × 20 cm, demonstrating the capability of the bicomponent blow spinning process to produce gradient textiles on a large scale. Figure 1c exhibits the versatility of producing GMFT in various sizes, with precise control over their thicknesses (Fig. S2). The GMFT exhibits outstanding mechanical strength, with an exceptional tensile strength of 4.0 MPa (Fig. S3) and the capability to withstand loads of up to 5 kg (Fig. 1d). Moreover, radiative cooling materials with superior weather resistance and self-cleaning properties are essential for their practical outdoor applications. In challenging outdoor settings, the accumulation of solid contaminants like dust particles on the surface of radiative cooling materials would diminish their solar reflectivity and infrared emissivity, significantly reducing their cooling efficiency [43, 44]. The GMFT exhibits outstanding hydrophobic characteristics, with a water contact angle of 137.4° on the PVDF-rich side, maintaining exceptional hydrophobicity even when exposed to various contaminated liquids (Fig. S4). The changes in the water contact angles on the PVDF-rich side within 0 to 10 min are shown in Fig. S5a, and the contact angle was slightly decreased. Due to the abundant micropores on the fabric surface, water gradually penetrated the textile pores induced by capillary forces, causing the wetting state to transition from composite contact to homogeneous contact based on the Wenzel equation, but the contact angle remained as high as 128.6°. Even after accelerated aging tests (240 h), the instantaneous water contact angle after 30 s reached 130.5°, as illustrated in Fig. S5b. By situating the GMFT on an inclined roof to replicate outdoor conditions and applying water droplets onto its surface, the self-cleaning property was attained (Fig. S5c). Leveraging its flexibility, the GMFT can be easily bent and folded (Fig. 1e). Figures 1f and S6 demonstrate that the direct deposition of GMFT on irregular surfaces is achieved through bicomponent blow spinning, catering to diverse cooling applications.

The diameter of microfibers in textiles and the pore structure formed by the overlapping assembly of microfibers are crucial factors that influence their solar scattering properties [45–47]. Hence, it is essential to achieve precise modulation of fiber diameters in the GMFT. Pure PVDF and PMMA microfiber textiles, with diameters ranging of 0.5–3.0 and 0.3–1.5 μm, respectively, can be fabricated by simply adjusting the concentration and viscosity of the respective polymers in the spinning solution (Figs. S1 and S8). The concentration range of the PVDF spinning solution was tailored between 10 wt% and 20 wt%. A lower concentration would lead to polymer entanglement, hindering fiber formation due to inadequate support for airflow traction in the high-speed spinning process, resulting in the formation of jet droplets. Conversely, a higher concentration impedes airflow traction in overcoming internal droplet tension, potentially causing nozzle clogging and resulting in non-uniform fiber diameters with numerous beads [48–51]. The spinning concentration range for PMMA is wider, spanning from 10 wt% to 25 wt%. The optimal morphology, characterized by a smooth microfiber surface and well-arranged overlapping pores, was attained when the mass fraction of PVDF was 15 wt%. Solar spectral reflectance measurements of these fibrous textiles, produced from various mass fractions of the PVDF spinning solution, revealed that the highest solar reflectance was achieved with a 15 wt% mass fraction of the PVDF spinning solution (Fig. S9). Consequently, the mass fraction of the PVDF spinning solution for the GMFT preparation was set at 15 wt%, and the mass fraction of the PMMA spinning solution was maintained at a similar concentration to ensure rapid and homogeneous mixing. By employing such a concentrated spinning solution, the diameters of the microfibers in PVDF and PMMA textiles, prepared from the two spinning inks, were 2.0 and 0.3 μm, respectively (Fig. S10), showing significant diameter differences conducive to designing a gradient in fiber diameter. Moreover, to facilitate bicomponent blow spinning, the miscibility of PVDF and PMMA in their solvent-free states was investigated. Infrared absorption spectra and differential scanning calorimetry (DSC) plots of polymer composites containing these two polymers at different ratios demonstrated their strong miscibility (Fig. S11), evident from the significant reduction of crystallization peaks of PVDF at 840 cm^−1^ and a redshift of the C = O bond of PMMA, and a single glassy transition temperature in the composites [52, 53]. SEM images of MFT_FA_ with varying PVDF/PMMA ratios of 7/3, 5/5, and 3/7, respectively, depicted noticeable changes in fiber diameters of the resultant microfiber textiles (Fig. S12). The microfiber diameters exhibited a gradual decrease from 1.5 to 0.5 μm as the PVDF content decreased, demonstrating efficient regulation of fiber diameters ranging from 0.3 to 2 μm by adjusting the components in the spinning solution. This observation underscores the straightforward creation of gradient fiber-diameter structures in the textiles.

**Fig. 2:**
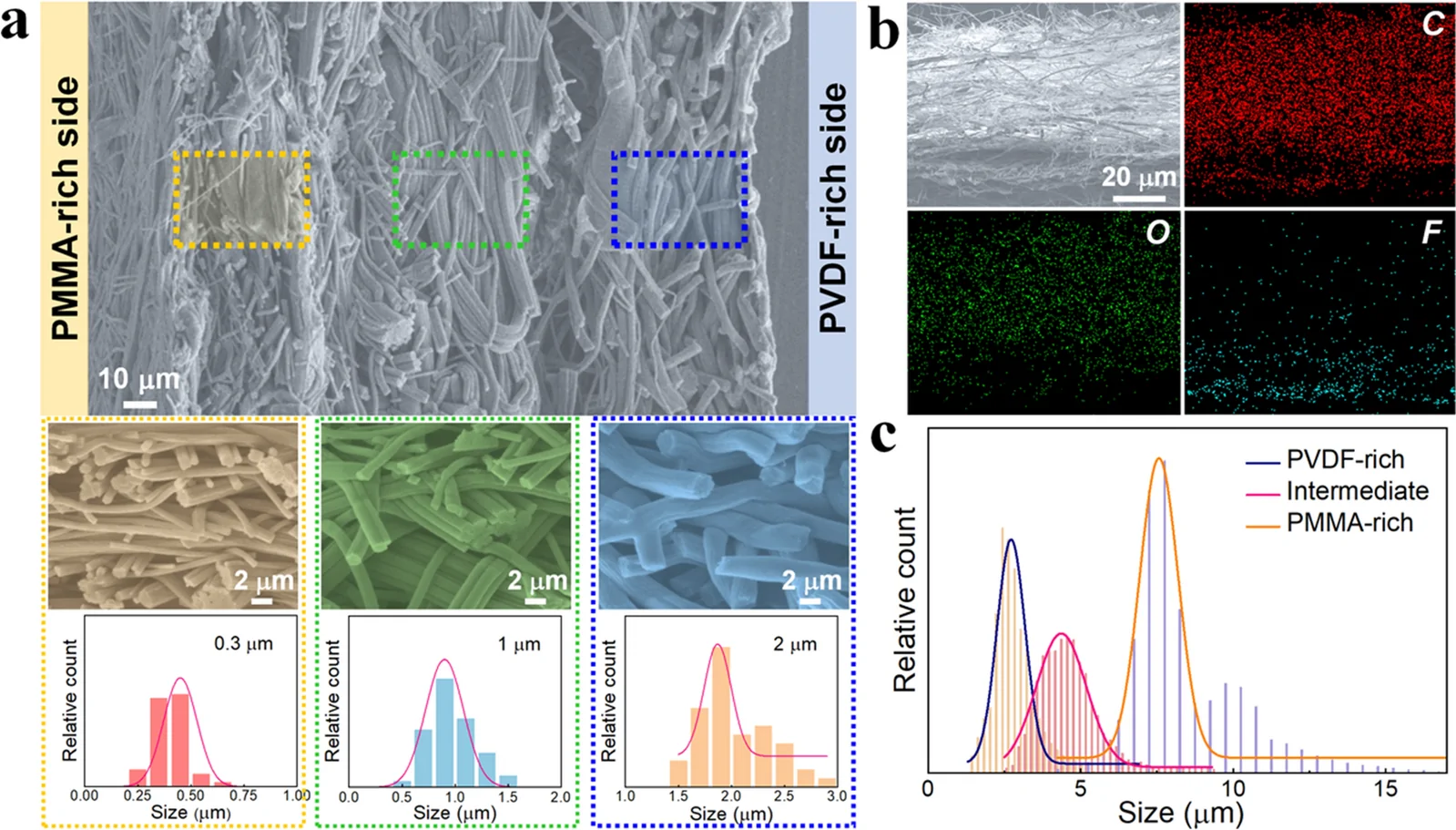
Characterization of dual-gradient structures of GMFT. **a** Cross-sectional SEM image displaying an overview of the GMFT, with representative SEM images and microfiber-diameter distributions at various locations. **b** Cross-sectional SEM–EDS image of the GMFT and elemental mappings of C, O, and F. **c** Pore size distributions of the GMFT at different locations

The cross-sectional SEM image of the GMFT clearly illustrates a fiber-diameter gradient (Fig. 2a), showing a decrease in fiber diameter from the PVDF-rich to the PMMA-rich side. As PVDF was progressively replaced by PMMA in the spinning solution, fiber diameters consistently decreased from 2.0 to 0.3 μm. Figure 2b presents SEM–EDS mappings of the entire textile cross section, revealing the presence of the F element exclusively in PVDF and not in PMMA. The gradient distribution of F elements vividly visualizes the chemical composition gradient of PVDF and PMMA within the cross section of the GMFT. Furthermore, PMI pore size analysis of the GMFT indicated that on the PVDF-rich side, pore sizes concentrated between 2.0 and 3.0 μm, while on the PMMA-rich side, they ranged from 7.0 to 9.0 μm (Fig. 2c). The pore size distribution of the GMFT fell between the PVDF-rich and PMMA-rich sides, displaying a broader distribution conducive to sunlight reflection. Additionally, bending experiments were conducted on gradient-structured and double-layer composite textiles. After 100 bending-releasing cycles, the double-layer composite textile exhibited noticeable delamination, whereas the gradient-structured textile maintained exceptional structural stability (Fig. S13).

### Asymmetric Spectral Emission Properties of the GMFT

**Fig. 3:**
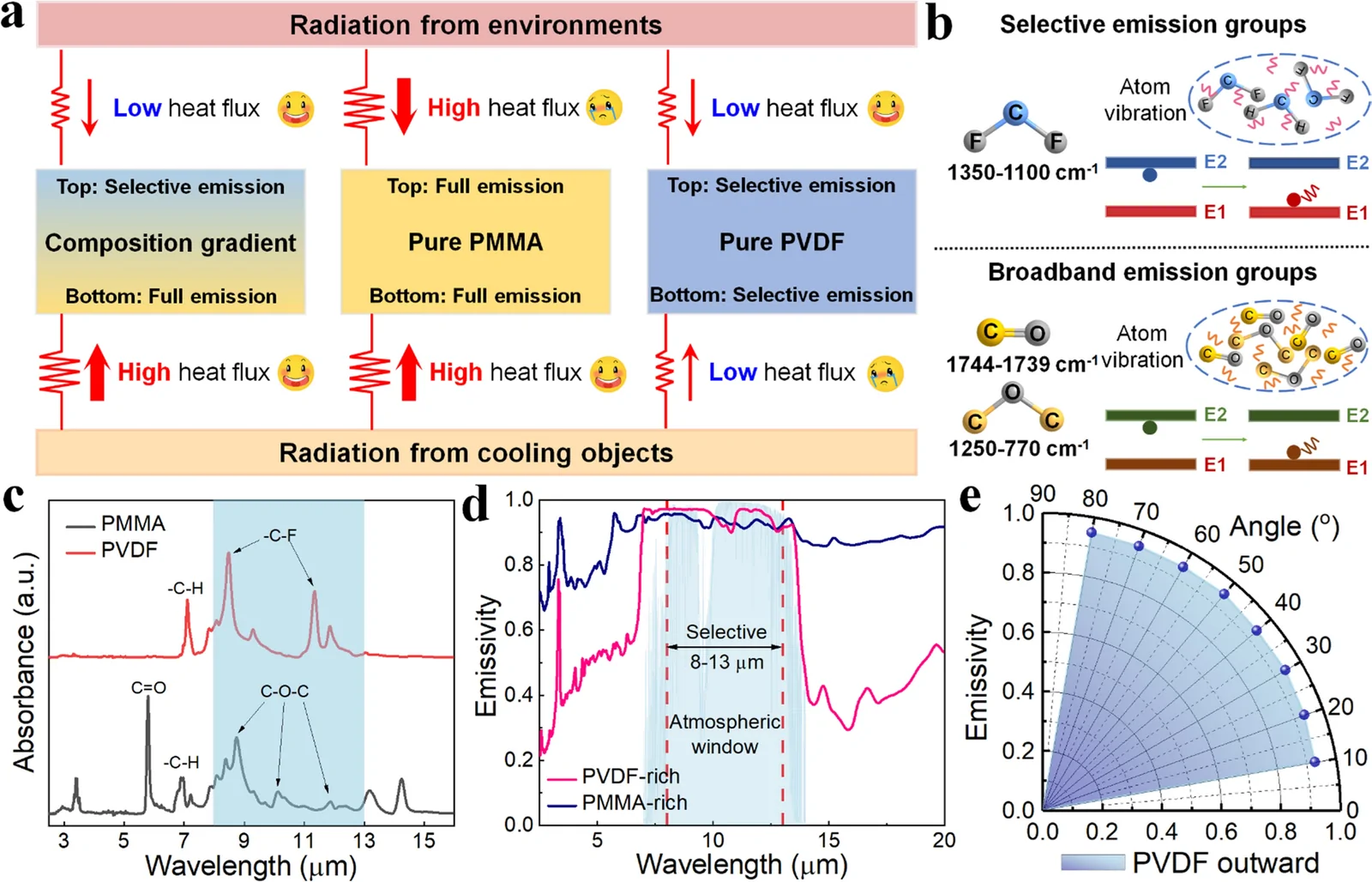
Working principle of radiative thermal-management GMFT with Janus spectrally selective emission surfaces. **a** Schematic comparing radiative heat exchanges using GMFT, pure PMMA textile, and pure PVDF textile in outdoor environments. **b** Selection of selective and broadband emission groups in PVDF and PMMA. **c** FT-IR spectra of PVDF and PMMA textiles. **d** Infrared emissivity of both sides of the GMFT with a thickness of 300 μm. **e** Average emissivity of the GMFT at emission angles ranging from 10° to 80°

Figure 3a depicts the working principle of the radiative thermal-management GMFT featuring Janus spectrally selective emission surfaces. The emission band of selectively emitting PVDF aligns with the atmospheric transparent window [9], which is less affected by ambient thermal radiation compared to the broad infrared spectrum-emitting PMMA [47]. In outdoor cooling systems, both selective and non-selective emitters can function as the low-temperature side, receiving a continuous stream of intense thermal radiation from high-temperature objects. While the selectively emitting PVDF efficiently emits infrared radiation within the atmospheric window, its selectivity limits the effective absorption of thermal radiation from neighboring objects outside the atmospheric transparent window. As a result, thermal radiation absorption within the internal space is not promptly converted into heat, leading to its accumulation and a significant reduction in the cooling effect. Therefore, it is crucial to establish asymmetric emission spectra on both sides of a radiative cooling material, where the outer side should emit the heat produced by thermal radiation captured on the inner side into the cold space. By opting for PVDF with high emission in the atmospheric transparent window as the outer layer and PMMA with broad-spectrum absorption as the inner layer, thermal radiation absorption from the inner side is guaranteed. The asymmetric spectral design of the gradient film of GMFT accommodates broad-spectrum high emission (high absorption) inward and high emission outward, thereby facilitating rapid heat dissipation.

When a polymer chain absorbs or releases energy, it triggers molecular movement, resulting in significant stretching and vibration of atoms. Photons are emitted when electrons shift from high to low valence bands, enabling the emission of thermal energy as electromagnetic waves. Conversely, photons are absorbed when electrons transition from low to high valence bands through radiation absorption. PVDF is characterized by abundant C-F bonds (1350–1100 cm^−1^), while PMMA is rich in C = O (1744–1739 cm^−1^) and C–O–C (1250–770 cm^−1^) bonds (Fig. 3b). Figure 3c reveals that PVDF exhibits distinctive absorption peaks of C-F and C-H bonds within the atmospheric transparent window, with minimal characteristic peaks outside this window, defining its selective emission characteristics. In contrast, PMMA displays abundant characteristic absorption peaks across the near- and mid-infrared spectrum, including C = O, C–O–C, and C-H bonds, indicating its potential for broad-spectrum absorption materials. These bonds emit strong thermal radiation, making PVDF suitable for selective infrared emission and PMMA ideal for broad-spectrum infrared absorption. Compared to bulk materials, microfiber-assembling porous structural textiles with high specific surface area also boost the thermal radiation capacity (Figs. S14 and S15). Figure 3d demonstrates that the GMFT exhibits a high mid-infrared emissivity on the PVDF-rich side, specifically 95.3% within the wavelength range of 8 to 13 μm. Conversely, the PMMA-rich side of the GMFT shows a broad infrared emissivity of 90.9% across the spectrum from 2.5 to 20 μm. We quantitatively reflected the differences of the emission spectra on both sides of the GMFT by calculating the surface selection ratio (*γ*). According to Formula 1, the *γ* is defined as the ratio of the average emissivity in the atmospheric window (8 to 13 μm) to the average emissivity in the non-atmospheric window (2.5–8 and 13–20 μm):

1 $$\frac{{\int }_{8\mu m}^{13\mu m} \varepsilon \left(\lambda \right)d\lambda /{\int }_{8\mu m}^{13\mu m} d\lambda }{\left({\int }_{2.5\mu m}^{8\mu m} \varepsilon \left(\lambda \right)d\lambda +{\int }_{13\mu m}^{20\mu m} \varepsilon \left(\lambda \right)d\lambda \right)/\left({\int }_{2.5\mu m}^{8\mu m} d\lambda +{\int }_{13\mu m}^{20\mu m} d\lambda \right)}$$

where ε(λ) is the emissivity at the wavelength λ. For the PVDF-rich side of the GMFT, the average emissivity in the 8 to 13 μm band is 95.3%, and the average emissivity in the 2.5 to 8 μm and 13 to 20 μm bands is 56.6%, with a selection ratio of 1.68. For the PMMA-rich side of the GMFT, the average emissivity in the 8 to 13 μm band is 93.2%, and the average emissivity in the 2.5 to 8 μm and 13 to 20 μm bands is 89.2%, with a selection ratio of 1.04. The infrared emissivity of both sides of the GMFT confirms the Janus spectral emission characteristics, enabling selective high emission outward and broad-spectrum absorption inward, thereby facilitating the dissipation of thermal radiation from internal heat sources to the external environment. Furthermore, the GMFT also maintains high emissivity across a wide range of angles in the atmospheric transparency window (Fig. 3e).

### Solar Reflectivity of the GMFT

**Fig. 4:**
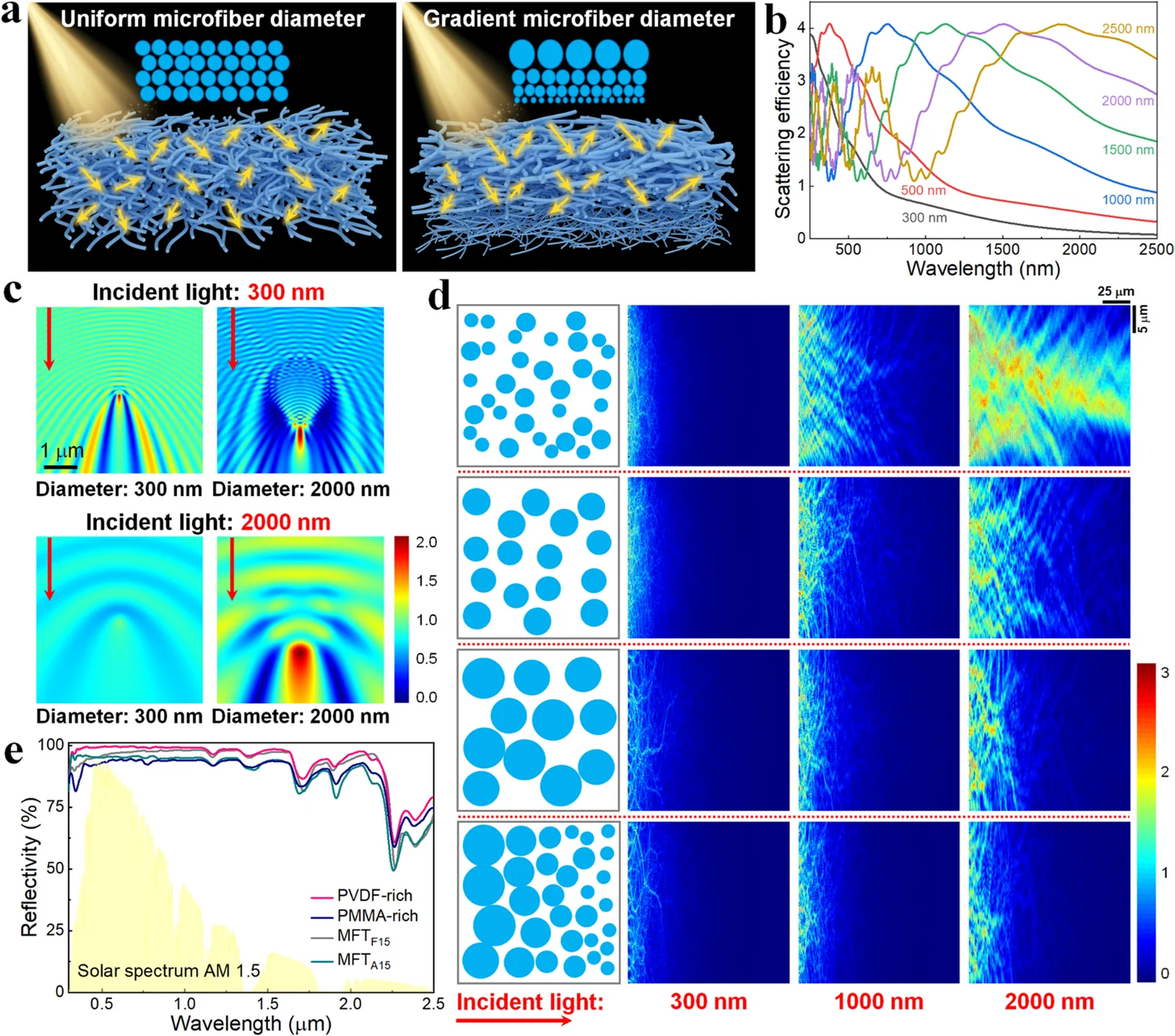
Solar reflectance performance of GMFT by gradient microfiber diameters. **a** Schematic illustrating the multiple Mie scattering of sunlight by textiles with uniform and gradient microfiber diameters. **b** Simulated scattering efficiencies of textiles with microfiber diameters ranging from 300 to 2500 nm across wavelengths of 250–2500 nm. **c** Near electromagnetic field distribution of textiles with microfibers of various diameters under incident light of 300 nm and 2000 nm. **d** Near electromagnetic field distribution of textiles with uniform (small, medium, and large) and gradient microfiber diameters under incident light of 300 nm, 1000 nm, and 2000 nm. **e** UV–Vis–NIR reflectance for GMFT, MFT_F15_, and MFT_A15_ within the solar wavelength range

To enhance radiative cooling power, it is essential to boost the reflectivity toward the sunlight. Abundant scattering units matching the 0.3–2.5 μm solar spectrum are highly desired. In comparison with a uniform structure, a variety of scattering units within the gradient structure, featuring pore sizes aligned with incident sunlight, diminishes the scattering depth within the material and reduces the frequency of scattering occurrences (Fig. 4a). Through the simulation of the scattering efficiency of spherical scattering units (Fig. S16) and cylindrical scattering units of varying sizes (Fig. 4b), it was observed that cylindrical scattering units exhibited enhanced scattering efficiency. The peak scattering efficiency varied with the fiber diameter, indicating that fiber textiles with a wide diameter distribution are more effective in achieving high efficiency in scattering sunlight within a confined scattering depth. Gradient-structured GMFTs, compared to uniform-structured porous materials, offer a wider pore size distribution and the potential for achieving a higher sunlight reflectance. The solar scattering mechanism using these micro-nanoporous structures was analyzed through MATLAB simulation (Fig. S17). Incident sunlight passing through the fiber–air interface results in robust scattering. Following the Mie scattering principle, light is scattered at a wide angle through the textile and re-scattered out of its surface via a complex path, thereby enhancing its overall reflectivity. The established scattering far-field and near-field irradiation electric field distribution reveals strong Mie scattering when the fiber diameter closely aligns with the incident light wavelength. For instance, when the incident light is 300 nm, a 300 nm fiber diameter exhibits a significant change in forward scattering angle, aiding the shorter scattering path of the incident light to escape the material surface. Conversely, when the incident light is 2000 nm, fibers of similar diameters undergo strong Mie scattering, resembling Rayleigh scattering due to their smaller size relative to the incident light wavelength. Electric field distributions also mirror substantial scattering effects. To visually demonstrate the scattering impact of fibers with diverse diameters on incident light of varying wavelengths, electric field distribution simulations of finite difference time domain (FDTD) were conducted (Fig. 4c). Notably, the scattering intensity is most pronounced in all directions when the incident light wavelength matches the fiber diameter. Figure 4d illustrates the electromagnetic simulation of electric field distribution in the nanofiber composite textile, categorizing fiber textiles into those with uniform-distributed diameters (ranging of 0.3–0.5 and 2.0–2.5 μm) and those with a continuous gradient distribution of diameters, generated through MATLAB simulations tapering from 2.5 to 0.3 μm. For a typical solar spectrum, by selecting three mission wavelengths (0.3, 1.0, and 2.0 μm), the electric field penetrates deeper in porous materials with uniformly distributed diameters compared to those with gradient distributed diameters. This suggests that these fiber textiles with the gradient structure are more conducive to efficiently scattering sunlight across a broad range within a limited depth and are more effective in blocking solar radiation. Figure 4e displays the solar reflectance for the prepared gradient-structured GMFT, the uniform-structured MFT_F15_, and the uniform-structured MFT_A15_. The GMFT, featuring a continuous gradient structure, exhibits superior solar reflectance compared to the other uniform structures. The solar reflectance of the PVDF-rich surface facing outward (98.7%) surpasses that of the PMMA-rich surface (93.8%). This difference primarily arises from the loosely arranged PMMA microfibers with a diameter of 300 nm, with relatively large pore sizes, resulting in reduced scattering capacity for long-wavelength sunlight and a consequent decrease in overall solar reflectance. The high whiteness of the GMFT is further corroborated by the CIE (Commission Internationale de l´Eclairage) chromaticity coordinates, confirming its exceptional solar reflectance (Fig. S18).

**Fig. 5:**
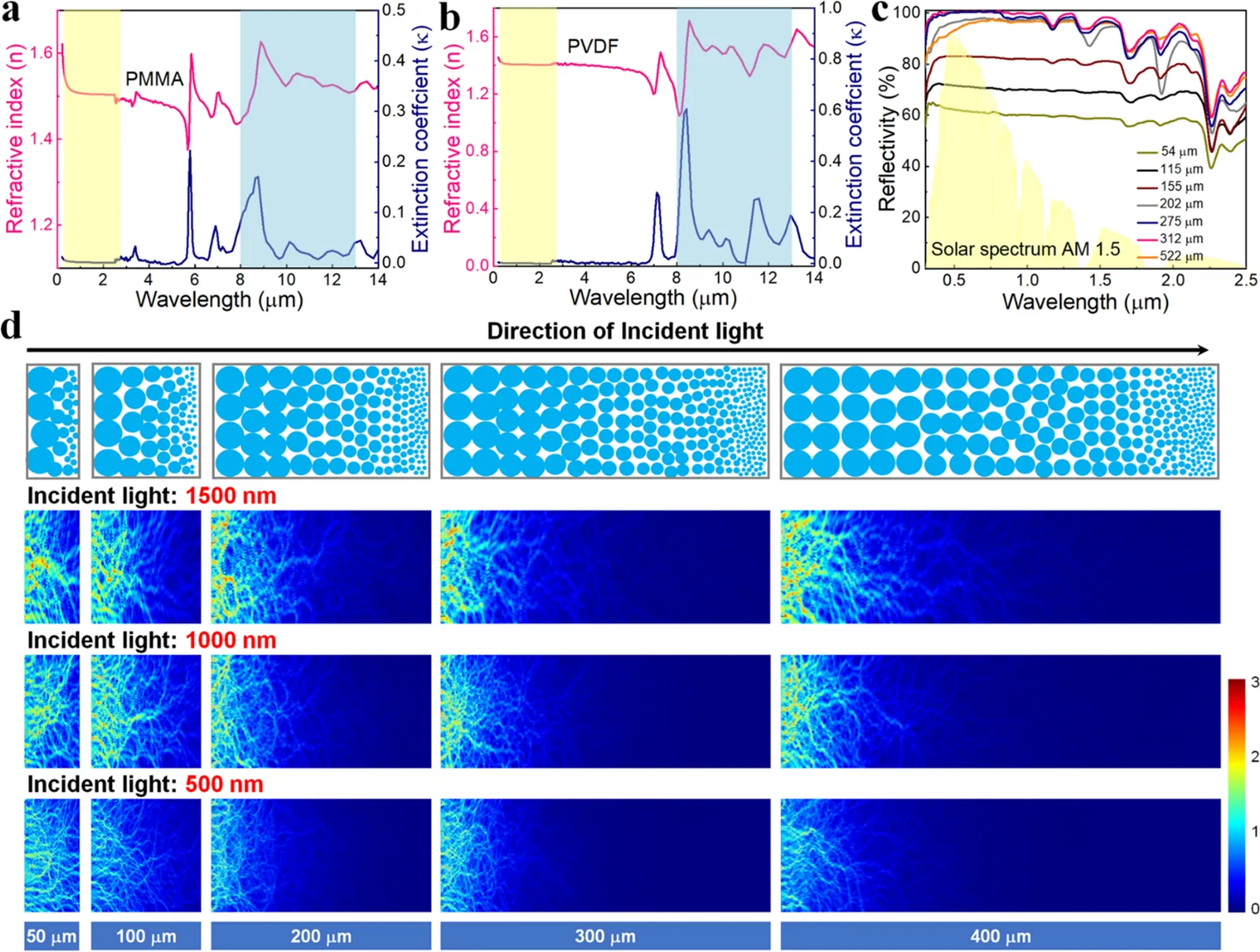
Thickness-dependent solar reflective properties of GMFT. Refractive index (*n*) and extinction coefficient (*k*) of **a** PVDF textile and **b** PMMA textile with a thickness of 300 μm. **c** UV–Vis–NIR reflectance for GMFT with various thicknesses in the solar wavelength range. **d** Near electromagnetic field distribution of GMFT with varying thicknesses under incident light of 1500 nm, 1000 nm, and 500 nm

The thickness-dependent spectral properties of the GMFT were investigated. Figure 5a, b demonstrates that the intrinsic PVDF and PMMA exhibit negligible extinction coefficients within the 0.3–2.5 μm wavelengths of the solar spectrum. Both polymers possess similarly high refractive indices (with a refractive index difference of 0.4 compared to air), leading to notable changes in refractive index at the polymer–air interface. This characteristic supports the potential for low absorption and efficient scattering of GMFT within the solar spectrum. The GMFT of varying thicknesses was then fabricated and evaluated for reflectance within the solar spectrum (Fig. 5c). Notably, sunlight reflectance fell below 90% for GMFT thicknesses ranging from 50 to 100 μm, peaking at 98.9% for a 300 μm-thick GMFT. However, reflectance decreased with further increases in the GMFT thickness. To explore the correlation between the thickness of the gradient structure and scattering efficiency, typical light waves in the solar spectrum (0.5, 1, and 1.5 μm) were selected to analyze the scattering depth of the continuous gradient structures with thicknesses of 50, 100, 200, 300, and 400 μm through electric field simulation analysis (Fig. 5d). The simulation revealed that the probing depth of incident light ranged from 100 to 200 μm regardless of the structural homogeneity. Excessive simulation length for the gradient structure diminishes its advantage of high reflection, while insufficient simulation fails to provide adequate scattering units and paths for light reflection. The gradient structure below 200 μm thickness lacks the necessary conditions to achieve efficient scattering of incident light, potentially leading to light transmission or incomplete reflection. The optimal performance was observed at a thickness of 200 μm for the gradient structure, with diminishing effectiveness beyond 400 μm due to a convergence toward uniformity in the detection depth range, resulting in reduced reflectivity. The trend of solar reflectance from actual GMFT samples aligned with FDTD simulations, underscoring that the GMFT with continuous gradient structures (0.3–2.5 μm) in the textile, around 300 μm thickness, achieves an optimal solar reflectivity, effectively mitigating solar radiative heat gain. To assess the UV resistance of the GMFT, accelerated aging tests were conducted under continuous UV irradiation. The GMFT exhibits high-durable stability by maintaining 97.9% of its initial solar reflectance following the accelerated UV aging tests (Fig. S19). This exceptional performance validates its superior resilience to photodegradation, a critical attribute for prolonged outdoor use.

### Outdoor Radiative Thermal-Regulating Performance of the GMFT

**Fig. 6:**
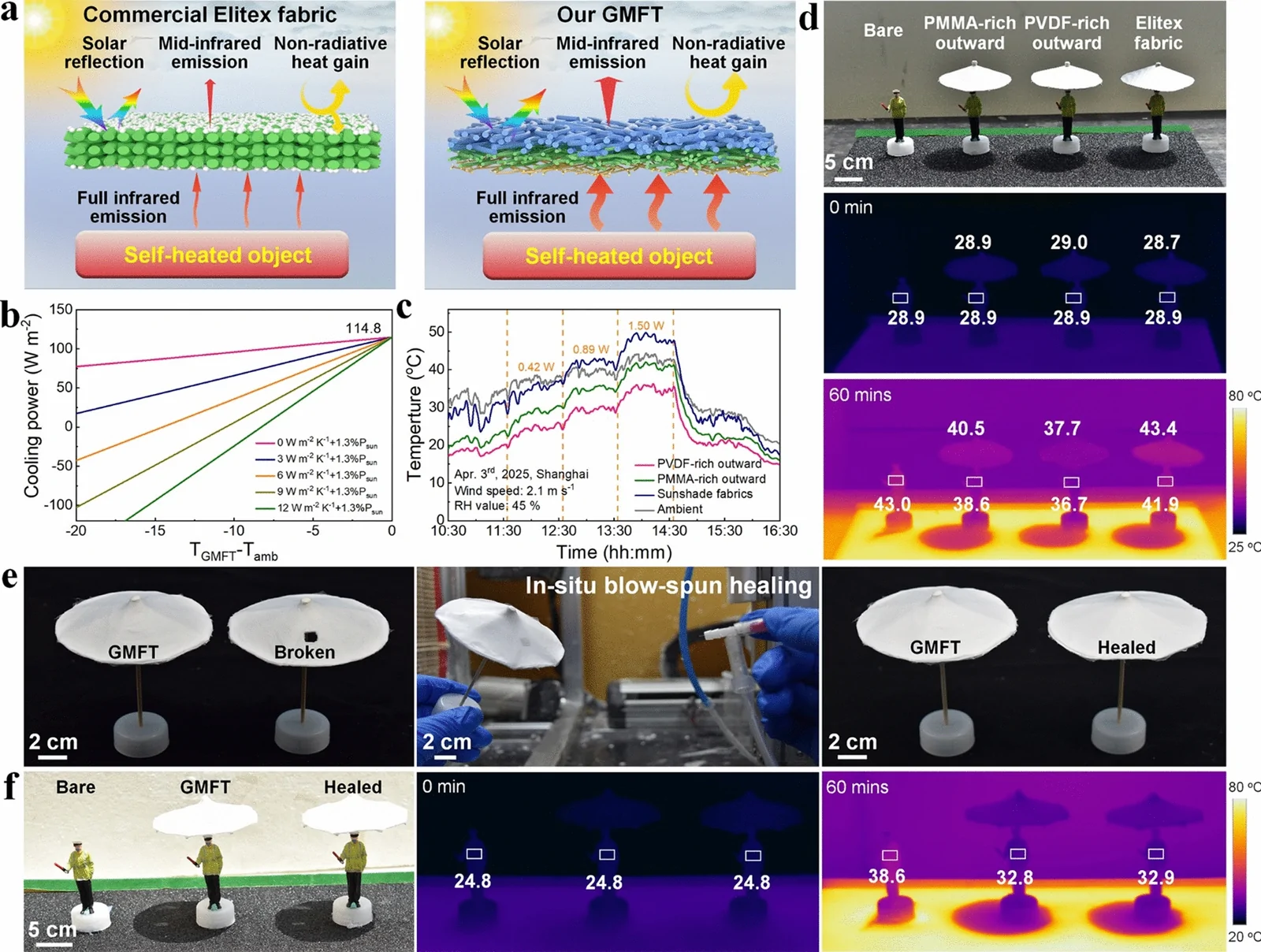
Radiative thermal regulation performance of GMFT. **a** Schematic comparing the working principles of thermal regulation using commercial Elitex fabrics and GMFT. **b** Calculated net daytime cooling power of GMFT. **c** Solar intensity and real-time temperatures of GMFT and Elitex fabrics for thermally regulating self-heated enclosed spaces. **d** Photograph and infrared images depicting the thermal profiles of a bare dummy model, and dummy models shaded by GMFT (with PMMA-rich outward, PVDF-rich outward) and Elitex fabrics before and after exposure to direct sunlight for 60 min. **e** Photographs showing in situ blow-spun healing of the broken GMFT. **f** Photograph and infrared images showing the thermal profiles of a bare dummy model, and dummy models shaded by pristine GMFT and broken-healed GMFT before and after exposure to direct sunlight for 60 min

Figure 6a elucidates the working concept of GMFT for radiative thermal management and distinguishes it from Elitex fabrics (commercial shading fabrics). Elitex fabrics amalgamate the functionalities of traditional shading fabrics with the highly reflective attributes of aluminum coating to fulfill solar energy reflection. Although the aluminum coating endows the fabric with high solar reflectance, it elevates the thermal conductivity of Elitex fabrics, hindering its efficacy in attenuating non-radiant heat gain from the surroundings. Concurrently, the low absorption and high reflection of metallic aluminum in the infrared spectrum further diminish their radiative cooling capabilities toward the atmosphere and its absorption of thermal radiation from internally heated spaces. However, the GMFT aims to revolutionize outdoor solar-thermal management by efficiently scattering sunlight and absorbing internal thermal radiation through a broader distribution of scattering units and asymmetric spectral characteristics compared to woven fabrics. This is achieved via porous textiles constructed through the gradient assembly of these microfibers with varying diameters. In contrast to Elitex fabrics, GMFT exhibits lower thermal conductivity (Fig. S20), heightened solar reflectance (Fig. S21), increased mid-infrared emissivity (Fig. S22), and enhanced infrared absorption within self-heated objects (Fig. S23). Figure 6b depicts the calculated daytime cooling power by using the GMFT. Assuming a solar radiation power of 1000 W m^−2^ and an ambient temperature of 25 °C, the integrated non-radiative heat coefficient is considered variable to account for diverse heat transfer mechanisms, including convection and conduction. Neglecting non-radiative heat transfer, theoretical calculations indicate a significant daytime cooling capacity of 114.8 W m^−2^, while nighttime cooling power reaches 127.8 W m^−2^ through the utilization of GMFT (Fig. S24). Under ambient cooling conditions, the convective heat transfer coefficients were determined to be 0, 3, 6, and 9. Theoretical projections indicate that even with a convective heat transfer coefficient (h) value of 9 W m^−2^ K^−1^, GMFT is capable of achieving a maximum temperature reduction of 10.1 °C. Moreover, the thermal diffusion process of Janus spectral structures, selective spectral structures, and broad-spectrum emission spectral structures was simulated against an internal 200 W m^−2^ ideal infrared radiation heat source under 800 W m^−2^ solar radiation and 30 °C atmospheric conditions using COMSOL. Monitoring the surface temperatures of these structures and the internal constant power heat source revealed that the Janus spectral structure exhibited the lowest temperature, 9.8 °C lower than the broad-spectrum structure (Fig. S25). Additionally, the selective spectrum was observed to create a significant spectral thermal resistance, impeding the transfer of thermal radiation from the interior heat source, resulting in a temperature of up to 65.3 °C for the same heating power. The broad-spectrum emission structure efficiently absorbed thermal radiation from within but did not effectively mitigate external thermal interference. In contrast, the heat source shielded by the Janus spectral structure maintained the lowest surface temperature of 58.6 °C, facilitating the efficient dissipation of thermal radiation from the internal heat source.

To evaluate the practical radiative cooling efficacy of GMFT, performance assessments were carried out under direct sunlight in hot conditions. A ceramic heating pad with adjustable heating power was incorporated into an insulated foam box to serve as a thermal radiation source, mimicking a scenario of cooling a self-heated space to emulate radiative cooling for self-heated objects (Fig. S26). In Fig. 6c, the temperature variations for both sides of the GMFT are compared with those of conventional sunshade fabrics. These measurements were carried out under an average solar irradiance of 900 W m^−2^, and the heating power was incrementally adjusted to 0.42, 0.89, and 1.50 W using a variable DC power supply, with each level maintained for 1 h (Fig. S27). Despite the internal environment being warmer than the surrounding ambient temperature due to the internal heat source, the asymmetric spectral properties and high solar reflectivity of the GMFT facilitate optimal radiative cooling on the PVDF-rich side, resulting in a temperature decrease of 13.6 °C at a heating power of 1.50 W compared to conventional sunshade fabrics. Additionally, the radiative cooling performance of GMFT was assessed in a confined space devoid of a self-heated source (Fig. S28), where GMFT with an outward PVDF-rich side exhibited superior performance, achieving a substantial temperature decrease of 7.8 in a 40 °C hot weather (Fig. S29). Importantly, the outdoor radiative cooling performance of the GMFT on cloudy days was also measured, and the textile still achieved the sub-ambient cooling of 4.8–6.3 °C on three different days (Fig. S30).

In simultaneous experiments conducted in Beijing (Fig. S31) and Hong Kong (Fig. S32) to assess the radiative cooling performance of GMFT, intriguing results emerged. Despite facing a high relative humidity of around 90% in Hong Kong, the PVDF-rich side of GMFT with a selective emission surface managed to achieve a temperature reduction of 5.6 °C. Conversely, the PMMA-rich side, influenced by its broad emission spectrum, experienced diminished radiative cooling efficacy due to atmospheric radiation interference, particularly noticeable in Beijing’s arid environment (relative humidity below 30%). In this drier setting, the radiative interference from the atmosphere was notably reduced. Consequently, in the low-humidity environment of Beijing, where relative humidity levels were below 30%, the atmospheric radiation interference weakened, enabling the GMFT to achieve a remarkable 8.7 °C decrease in temperature through radiative cooling. An infrared thermography-based comparative analysis was conducted to visualize the radiative cooling performance of the GMFT against commercial sunshade fabrics. Figure 6d illustrates the practical application of GMFT and commercial sunshade fabrics in the form of sunshade umbrellas. Using an infrared thermal imaging camera, the temperatures of model umbrellas and mannequins under the sunshade were monitored during summer on an asphalt road. After 60 min of sunlight exposure, GMFT exhibited the smallest temperature increase (10.8 °C), a noteworthy 5.7 °C lower than the commercial sunshade fabric. Furthermore, the mannequin sheltered by the GMFT umbrella experienced a temperature 5.2 °C lower than that under the commercial material. Infrared thermography monitoring of various awning and tent usage scenarios (Figs. S33 and S34) consistently demonstrated its superior radiative thermal-management performance. The optical images presented in Figs. 6e and S35 illustrate the in situ blow-spun healing capabilities of the GMFT when breakage occurs during usage. Following healing, the spectroscopic analysis of the restored GMFT revealed that it retained outstanding solar reflectance and infrared asymmetric spectral characteristics (Fig. S36). Subsequent infrared thermography assessments confirmed that the repaired GMFT continued to exhibit exceptional radiative cooling performance (Fig. 6f). Furthermore, the hydrophobicity of the healable GMFT was evaluated, revealing a consistent contact angle of 136.1°, indicating enduring high self-cleaning effectiveness during water droplet assessments (Fig. S37). Subsequently, the mechanical properties of the healable GMFT were examined (Fig. S38), demonstrating that its tensile strength was largely preserved with highly stable mechanical properties. These findings underscore the versatile applications of GMFT in diverse settings, including outdoor shading products, logistics, and transportation, indicating its potential for widespread commercial utilization.

## Conclusions

In summary, a large-area, mechanically flexible, and healable microfiber textile with a dual gradient in chemical composition and fiber diameter is fabricated through bicomponent blow spinning. The chemical composition gradient in this textile results in a unique Janus infrared-absorbing surface. The textile with the fiber-diameter gradient exhibits a hierarchically porous sunlight reflection interface, enabling a competitive solar reflectivity of 98.7% on its outer surface. Moreover, the outer surface of the textile is enriched with highly emissive C-F groups in the atmospheric window, leading to a mid-infrared emissivity of 95.3%, while the inner surface displays a broad infrared absorptivity of 90.9% facilitating efficient radiative heat exchange with the underlying self-heated objects. These characteristics enabled by the dual-gradient structures make the textile highly efficient in facilitating radiative heat transfer with adjacent thermal-emitting objects, thereby enabling the radiative cooling of conventional unheated objects and self-heated objects that are warmer than the surrounding environment. As a demonstration, this textile exhibits an average outdoor cooling of unheated and self-heated objects by 7.8 and 13.6 °C, respectively, outperforming commercial Elitex sunshade fabrics. This bicomponent blow spinning approach also endows the textile with exceptional structural healing and restoration capabilities. In cases where the textile has significant holes due to extended use, complete restoration of both structural integrity and cooling functionality is achieved through in situ blow-spun healing. This study introduces an innovative concept in fabricating dual-gradient structural cooling textiles for multi-scenario radiative cooling of outdoor objects and individuals.

## Supplementary Information

Below is the link to the electronic supplementary material.

Supplementary file1 (DOCX 8946 KB)
