# Supplementary material for: Scalable and Healable Gradient Textiles for Multi-Scenario Radiative Cooling via Bicomponent Blow Spinning
Source: Nanomicro Lett. 2025 Dec 5;18:86. doi: 10.1007/s40820-025-01947-2 (PMC12678696; doi:10.1007/s40820-025-01947-2)
Supplement: Supplementary file 1 — Supplementary file1 (DOCX 8946 KB) [file 40820_2025_1947_MOESM1_ESM.docx]

Supporting Information for

**Scalable and Healable Gradient Textiles for Multi-Scenario Radiative Cooling via Bicomponent Blow Spinning**

Baiyu Ji^1^, Yufeng Wang^1^, Ying Liu^3^, Yongxu Zhao^1^, Fankun Xu^1^, Jian Huang^1^, Yue-E Miao^1^, Chao Zhang^1^*, Tianxi Liu^2^

^1^ State Key Laboratory of Advanced Fiber Materials, College of Materials Science and Engineering, Donghua University, Shanghai 201620, China

^2^ Key Laboratory of Synthetic and Biological Colloids, Ministry of Education, School of Chemical and Material Engineering, Jiangnan University, Wuxi 214122, China

^3^ Department of Materials Science and Engineering, City University of Hong Kong, 83 Tat Chee Avenue, Hong Kong, China

*Corresponding author. E-mail: czhang@dhu.edu.cn (Chao Zhang).

**S1. Characterization**

*Measurements of radiative cooling performance:* The radiative thermal regulation performance of the fabrics was evaluated at Donghua University, Shanghai, China (east longitude: 121^o^21'; northern latitude: 31^o^06'). A customized thermal-insulating enclosure, comprising an insulating foam box wrapped in aluminum foil, was employed without encasing the textile samples in a transparent low-density polyethylene film for insulation sealing. This approach highlights the benefits of harnessing our porous radiative cooling textiles, emphasizing their inherent low parasitic heat gains. Additionally, another insulation foam was positioned at the bottom of the enclosure to mitigate thermal conduction from the ground. The temperatures of the cooling samples (10 cm × 10 cm × 300 μm) and the surrounding environments were continuously monitored using A8-channel K-type thermocouples (Ilitech Co., Ltd. China). These thermocouples were connected to a computer running Picolog software for data logging at 10-second intervals. Carefully positioned at the base of the cooling samples with slight compression to guarantee full contact, the thermocouples facilitated accurate temperature readings. Each sample underwent a minimum of three measurements, and the average values were recorded for subsequent analysis.

*Measurements of the outdoor radiative cooling performance:* The radiative thermal regulation performance of the textiles was assessed at Donghua University, Shanghai, China (longitude: 121^o^21'; latitude: 31^o^06'). The GMFT required for testing was in-situ blow spun on a model sunshade framework, and commercial sunshade fabric was directly affixed to another framework. A custom miniature replica of an outdoor asphalt road was utilized to simulate real outdoor conditions where GMFT would be deployed, emphasizing the impact of intense thermal radiation from the hot ground during summer. Four identical mannequins were positioned, with three sheltered under the prepared sunshading fabric umbrellas. Subsequently, the models were exposed to direct summer sunlight for 60 minutes, and the cooling efficacy of both the GMFT and the commercial fabric was captured using an infrared thermography camera.

*Simulations of the solar scattering efficiency by FDTD*: The simulation was performed using the commercial software Lumerical FDTD Solutions, employing the FDTD method based on Maxwell's equations. A three-bit fiber cylinder model was positioned at the center of a rectangular air background, surrounded by perfectly matched layers (PML) boundaries (Fig. S38a, Supporting Information). Throughout the FDTD simulation, the grid size of the model was set to 50 nm. The incident light wavelength ranged from 0.3 μm to 2.5 μm, with the FDTD monitor tracking the scattering efficiency. The FDTD analysis yielded the near electromagnetic field distribution of fibers with varying diameters under different incident light conditions, and their respective scattering efficiency profiles were normalized using MATLAB. Due to computational constraints, a two-dimensional model was adopted. The creation of two-dimensional gradient-structured fiber membranes was accomplished using the MATLAB programming language (Fig. S38b, Supporting Information). A Total Field Scattered Field (TFSF) source, guided by Mie theory, was employed to assess the scattering efficiencies of adjustable gradient and uniform structures under specific incident light conditions and to determine the corresponding near electromagnetic field distribution.

*Measurements of the accelerated aging performance:* According to the ASTM G154-23 standard, accelerated aging tests of the textile were conducted using a QUV/Spray tester (Q-Lab Corporation, America), with each cycle consisting of 8-hour UV radiation (UVA-340, intensity of 0.89 J m^-^² nm^-1^) at 60 ^o^C. After the UV irradiation process, a 15-minute water spray was applied with a spray speed of 7 Lpm (water pressure 3bar), followed by 3.75 hours of condensation at 50 ^o^C, and a total of 20 cycles were performed.

*Simulation for thermal diffusion in textiles:* The thermal transfer simulations were conducted using the COMSOL Multiphysics software. 3D models were utilized to simulate the thermal diffusion mechanism of GMFT. The heat conduction simulations were confined to a square region measuring 120 mm * 120 mm * 120 mm and covered by textiles (Fig. S25a). A constant power infrared thermal radiation heat source of 200 W m^-2^ was positioned at the bottom of the monitored square space, while the other boundaries were set as adiabatic (with an emissivity value of 0.1). The ambient temperature was set to 303.15 °C, the outer space temperature was set to 3 K, and the solar thermal radiation power was designated as 800 W m^-2^. The emissivity of the simulated textiles can be found in Fig. S40.

**S2. Supplemental tables**

**Table S1.** Formulation of the various spinning solution for blow spinning.

| Sample | Polymer  [g] | MA  [g] | DMF  [g] |
| --- | --- | --- | --- |
| MFT_A10_ | 5.23 | 18.64 | 28.44 |
| MFT_A15_ | 8.31 | 18.64 | 28.44 |
| MFT_A20_ | 11.77 | 18.64 | 28.44 |
| MFT_A25_ | 15.69 | 18.64 | 28.44 |
| MFT_F10_ | 5.21 | 27.96 | 18.96 |
| MFT_F15_ | 8.28 | 27.96 | 18.96 |
| MFT_F20_ | 11.73 | 27.96 | 18.96 |
| MFT_F25_ | 15.64 | 27.96 | 18.96 |

**Table S2.** Formulation of the two spinning solutions for bicomponent blow spinning fabrication of MFT_F7A3_, MFT_F5A5_, and MFT_F3A7_.

| Sample | MFT_A15_ spinning solution [g] | MFT_F15_ spinning solution [g] |
| --- | --- | --- |
| MFT_F7A3_ | 15.0 | 35.0 |
| MFT_F5A5_ | 25.0 | 25.0 |
| MFT_F3A7_ | 35.0 | 15.0 |

**S3. Supplementary figures**


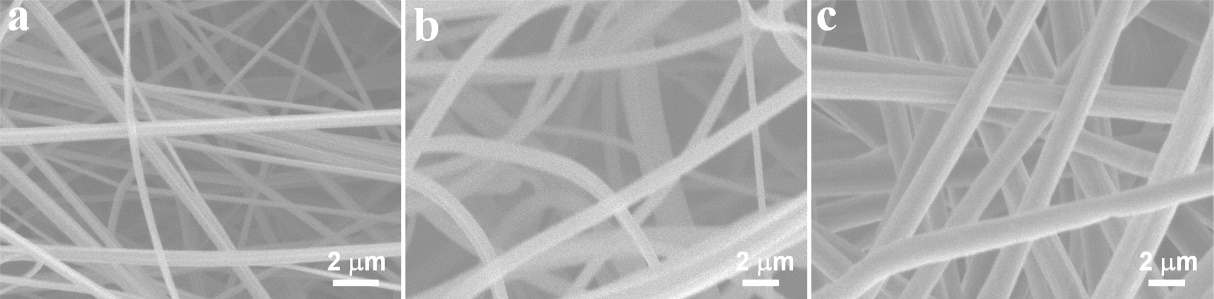


**Fig. S****1** SEM images of the bottom, medium, and top layers in the obtained GMFT.


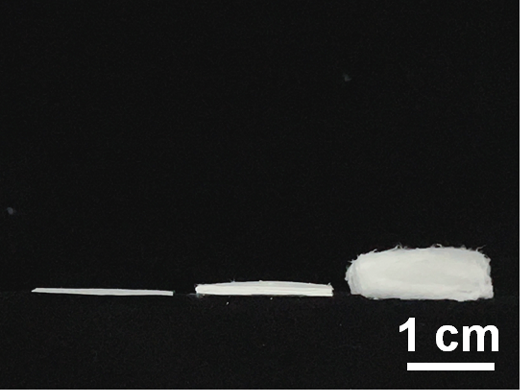


**Fig. S2** Photograph of the as-obtained GMFT with varying thicknesses.


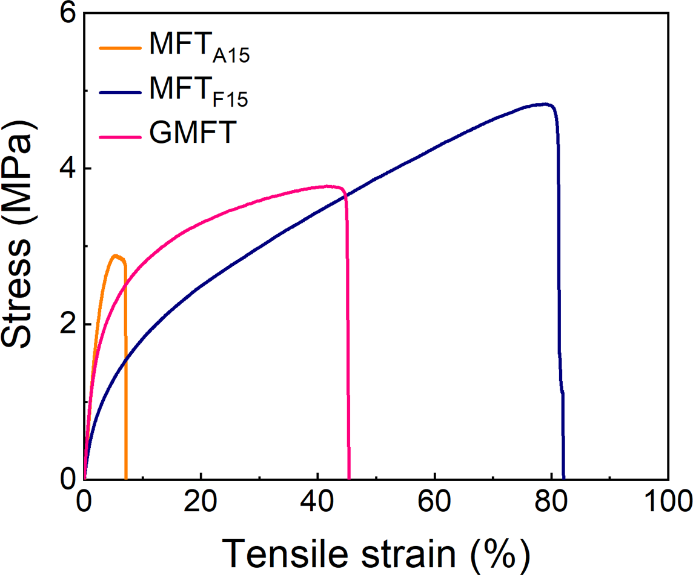


**Fig. S3** Stress-strain curves of MFT_A15_, MFT_F15_, and GMFT.


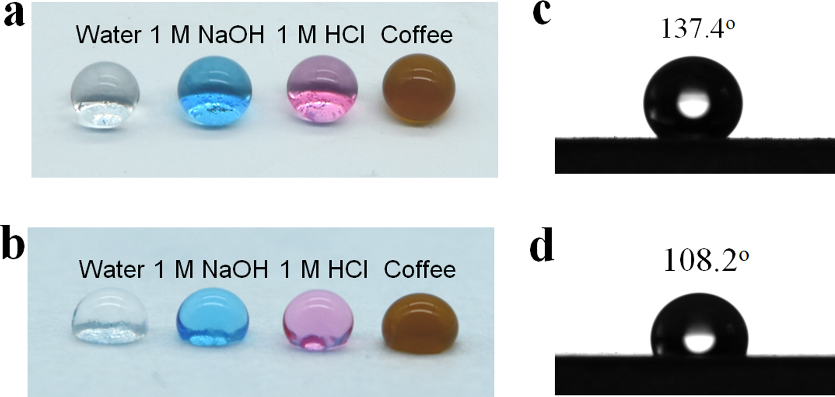


**Fig. S4** Photographs of various solutions on the surface of a) PVDF-rich and b) PMMA-rich sides of GMFT. Water contact angles of c) PVDF-rich and d) PMMA-rich sides of GMFT.


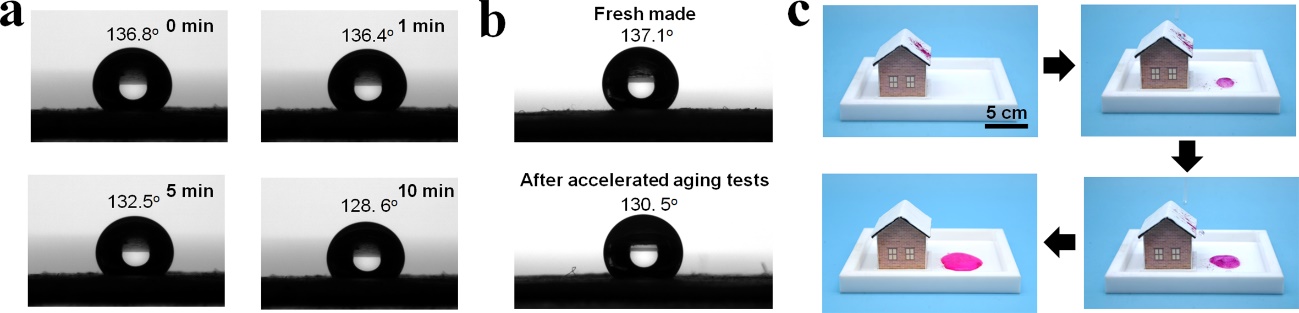


**Fig. S5** a) Water contact angles of the PVDF-rich side of the GMFT from 0 to 10 minutes. b) Variations in the water contact angles of the PVDF-rich side of the GMFT before and after the accelerated aging tests. c) Photographs demonstrating the self-cleaning capability of a house model with the GMFT roof. The GMFT roof exhibited rapid and efficient dye removal with minimal water washing, mimicking the practical elimination of surface dirt.


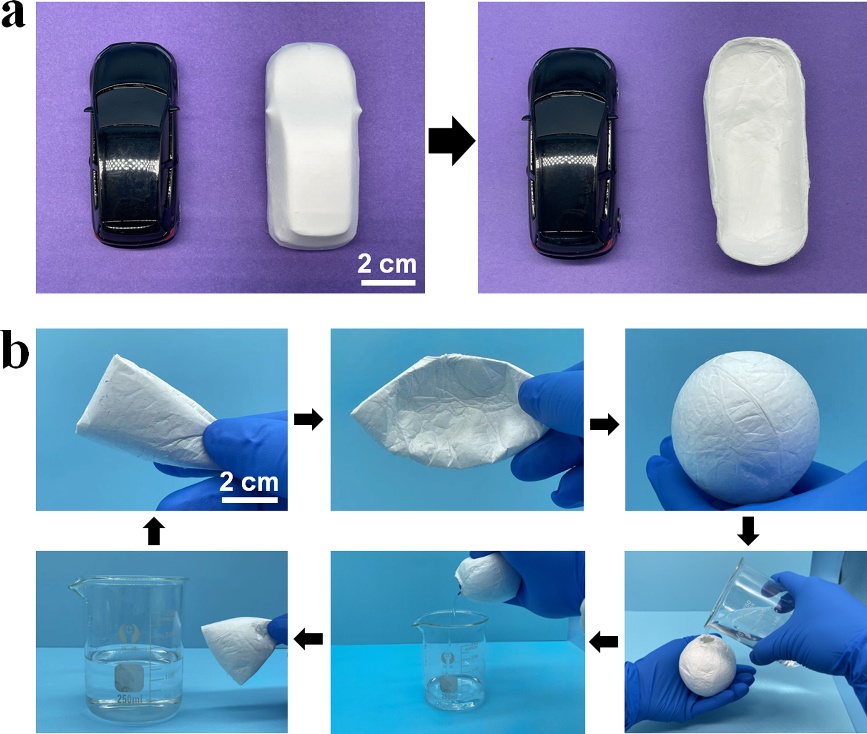


**Fig. S6** a) Photographs of the bicomponent blow spinning coating applied to the surface of a car model. Left: In-situ coating of the GMFT on the car model; Right: Coating after being peeled off from the car model. b) Photograph of the bicomponent blow spinning coating on a hollow sphere model, demonstrating the fabrication of a hollow GMFT sphere. The coating exhibits excellent hydrophobicity, allowing the sphere to retain a full cavity of water, along with notable flexibility.


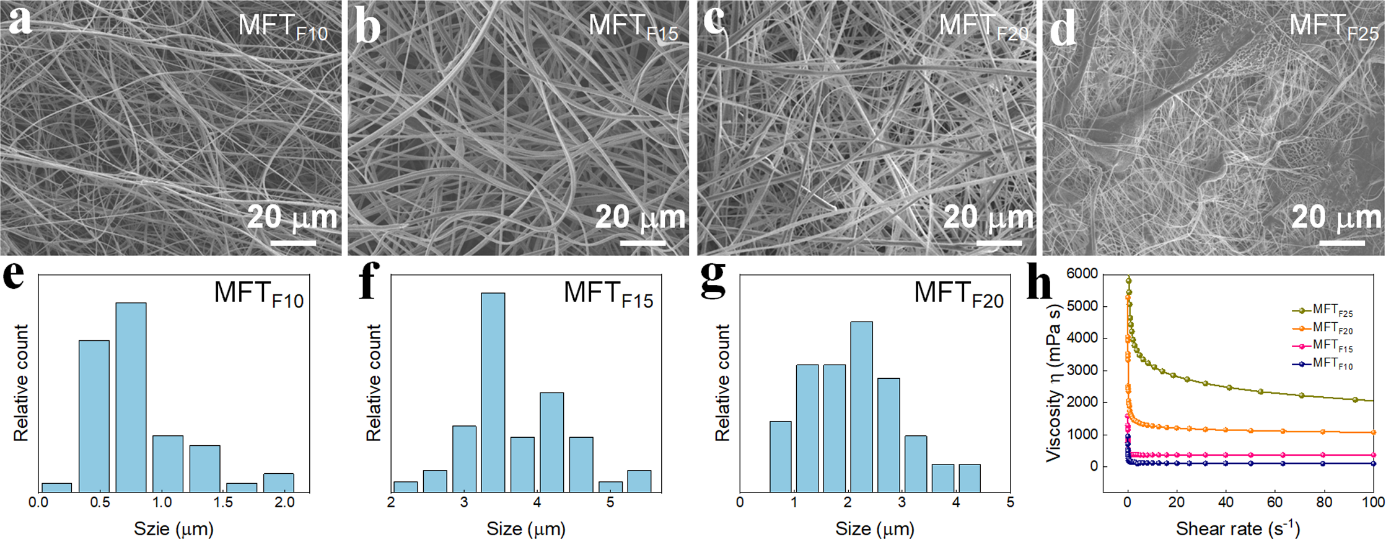


**Fig. S7** SEM images of a) MFT_F10_, b) MFT_F15_, c) MFT_F20_, and d) MFT_F25_. Size distributions of microfibers in e) MFT_F10_, f) MFT_F15_, and g) MFT_F20_.h) Viscosity of the PVDF spinning solution at various PVDF concentrations, measured across varying shear rates.


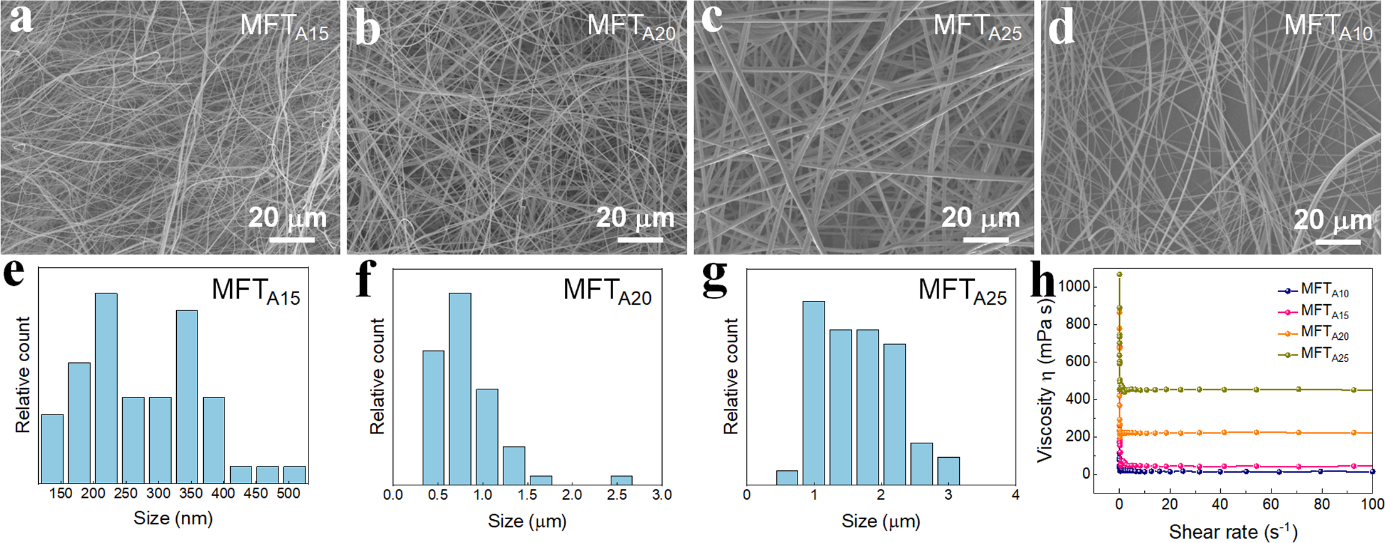


**Fig. S8** SEM images of a) MFT_A15_, b) MFT_A20_, c) MFT_A25_, and d) MFT_A10_. Size distributions of microfibers in e) MFT_A15_, f) MFT_A20_, and g) MFT_A25_.h) Viscosity of the PMMA spinning solution at various PMMA concentrations, measured across varying shear rates.


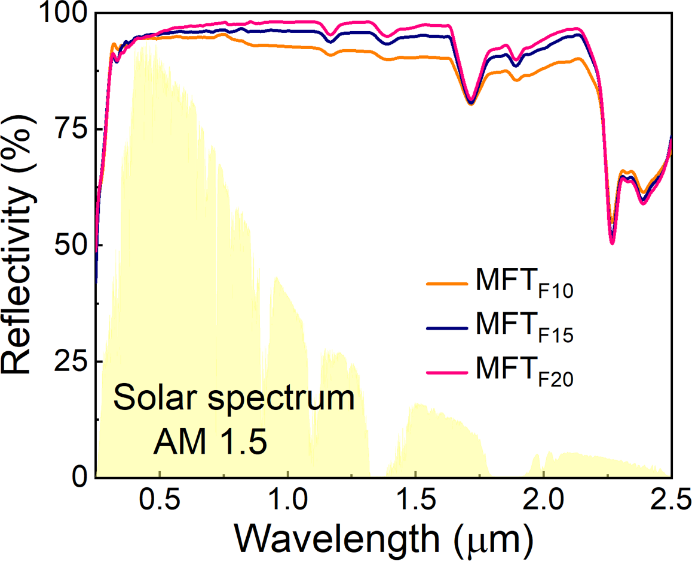


**Fig. S9** UV-vis-NIR reflectance measurements for MFT_F10_, MFT_F15_, and MFT_F20_.


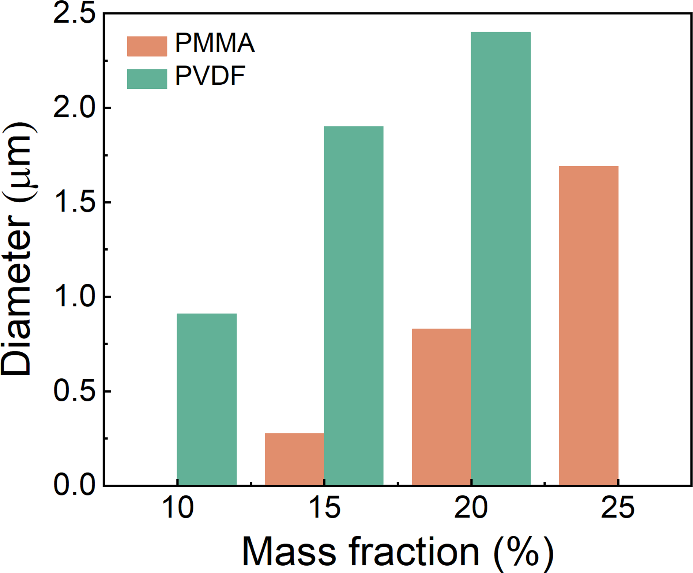


**Fig. S10** The average fiber diameter of PMMA and PVDF microfiber textiles produced from spinning solutions of varying concentrations.


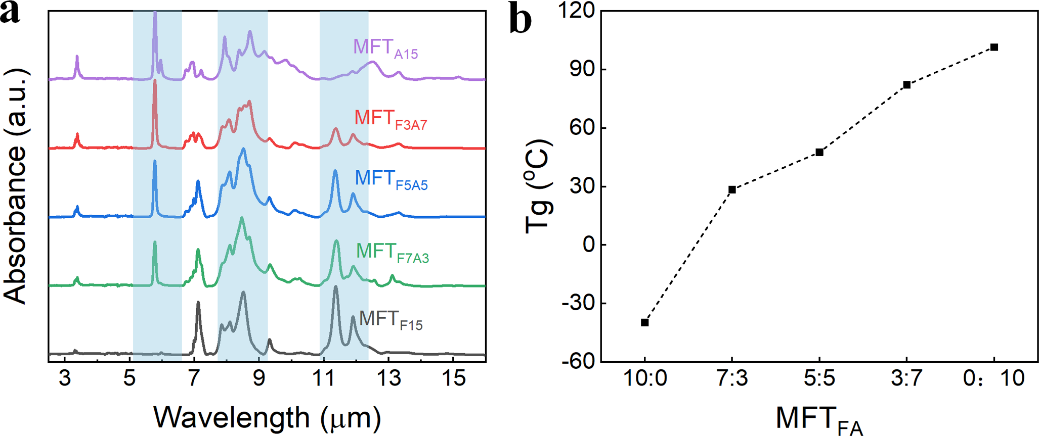


**Fig. S11** a) FITR spectra and b) glassy transition temperatures of MFT_A15_, MFT_F3A7_, MFT_F5A5_, MFT_F7A3_, and MFT_F15_.


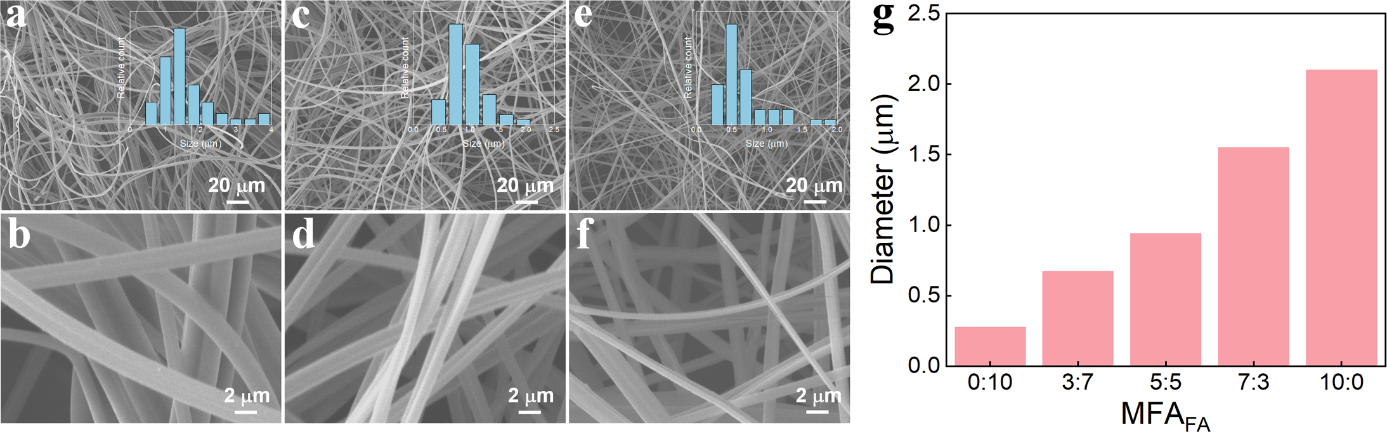


**Fig. S12** SEM images of a,b) MFT_F7A3_, c,d) MFT_F5A5_, and e,f) MFT_F3A7_ at low and high magnifications. Insets in a-c) are microfiber diameter distributions of the corresponding textiles. g) Average diameters of microfibers in MFT_F15_, MFT_F7A3_ , MFT_F5A5_, MFT_F3A7_, and MFT_A15_.


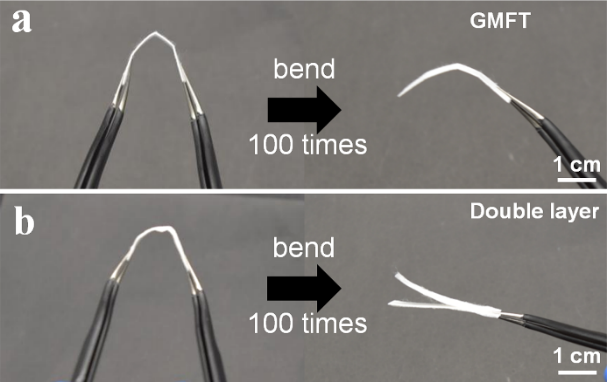


**Fig. S13** Photographs of a) the GMFT and b) the comparative double-layer PVDF-PMMA microfiber textile subjected to bending tests for 100 cycles.


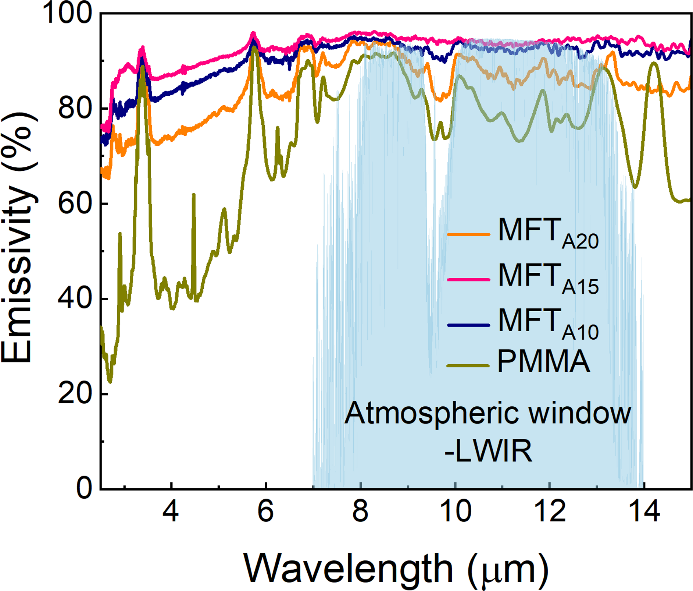


**Fig. S14** Emissivity for MFT_A20_, MFT_A15_, MFT_A10_ and pure PMMA compact film.


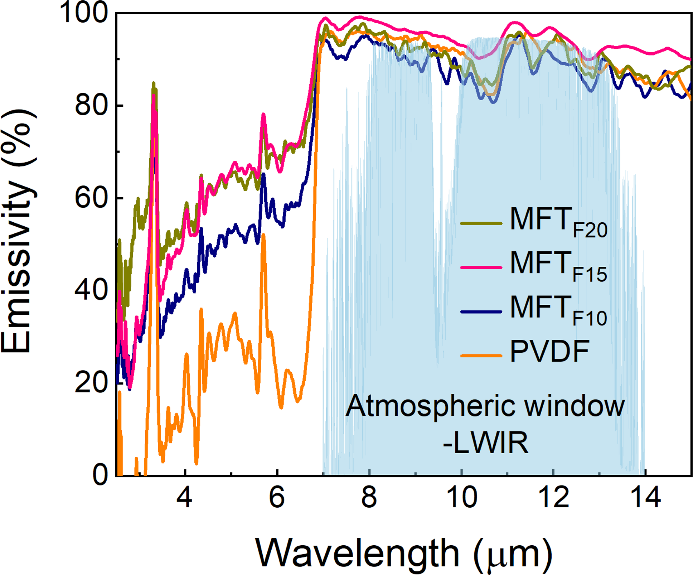


**Fig. S15** Emissivity for MFT_F20_, MFT_F15_, MFT_F10_ and pure PVDF compact film.


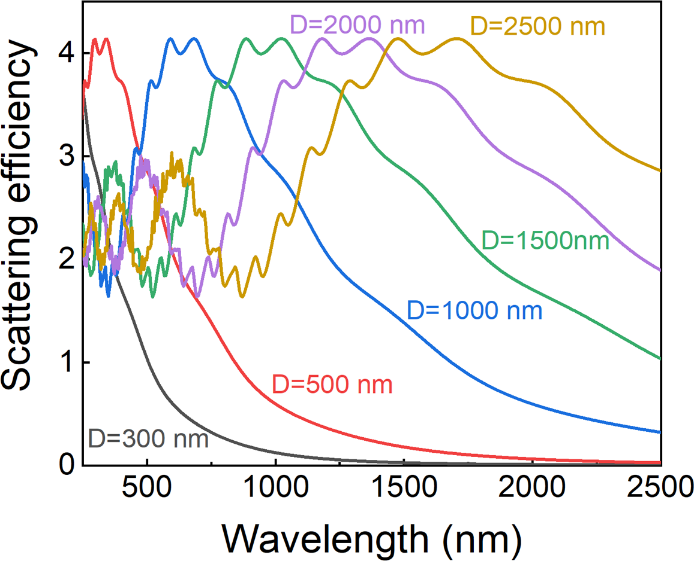


**Fig. S16** Simulated scattering efficiencies of spherical samples with sizes ranging from 300 to 2500 nm across wavelengths of 250 to 2500 nm.


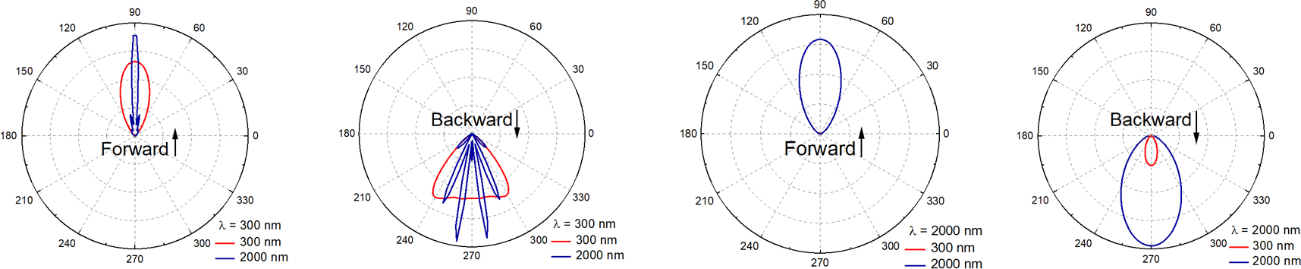
**Fig. S17** Polar diagrams illustrating the scattering direction distribution of porous structures with varying pore sizes (300 and 2000 nm) at specific incident wavelengths of 300 nm and 2000 nm.


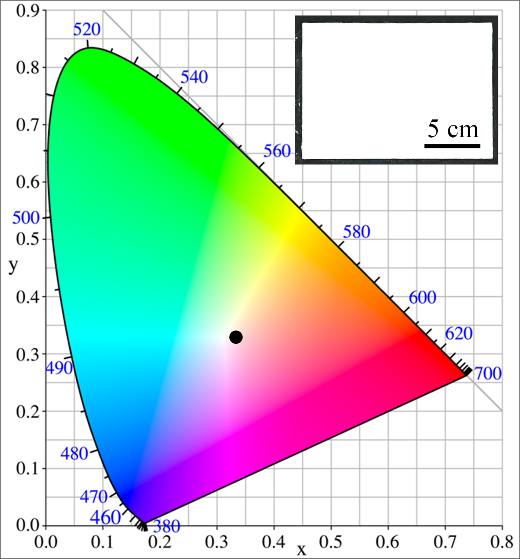


**Fig. S18** The CIE chromaticity coordinate of GMFT. Inset showing the photograph of GMFT.


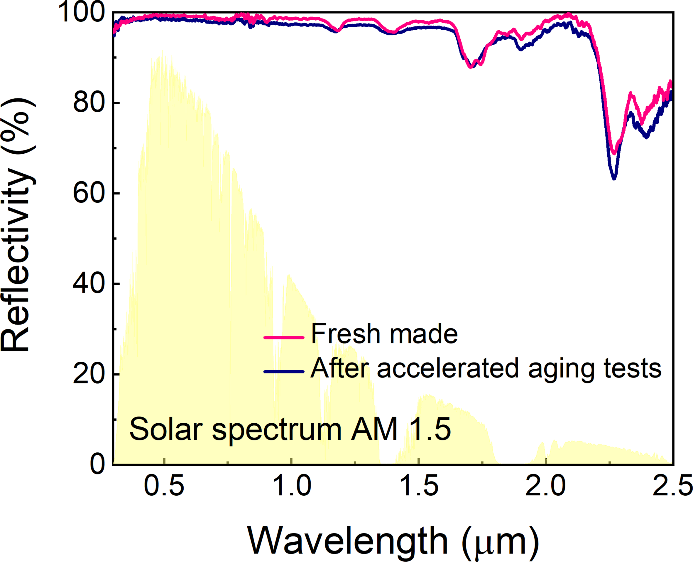


**Fig. S19** Reflectivity spectra of the fresh-made GMFT and the GMFT after the accelerated aging tests.


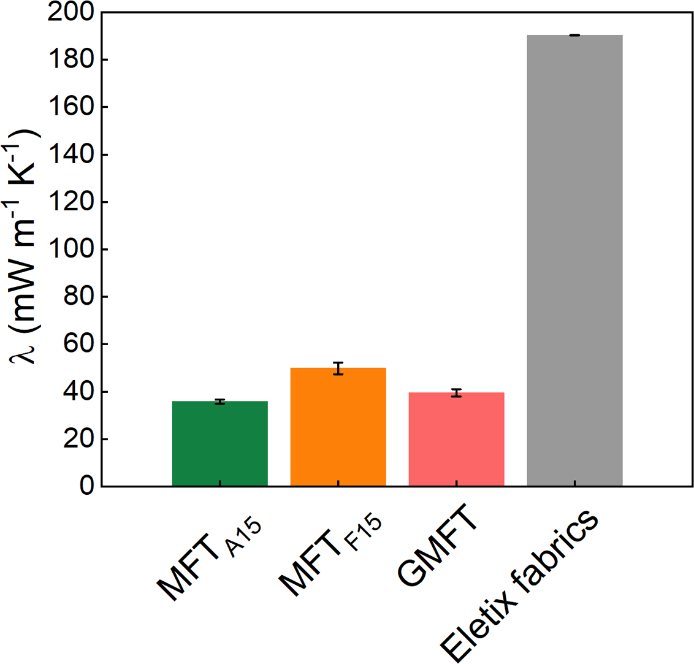


**Fig. S20.** Thermal conductivities of MFT_A15_, MFT_F15_, GMFT, and Elitex fabrics.


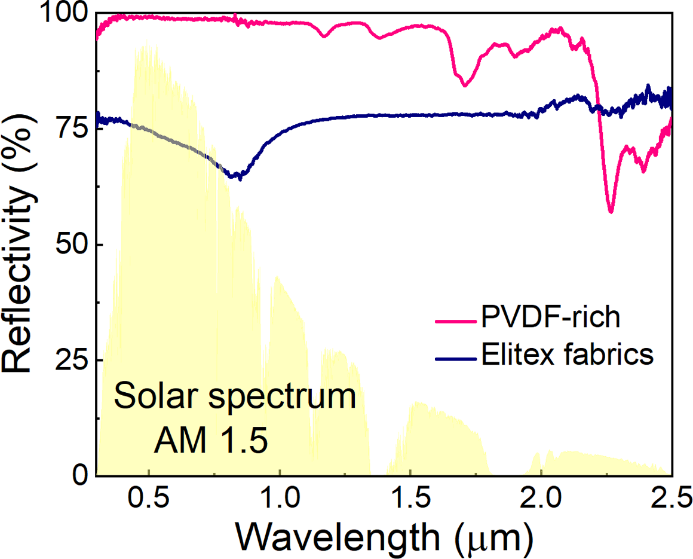


**Fig. S21.** UV-vis-NIR reflectance for the PVDF-rich side of GMFT and Elitex fabrics.


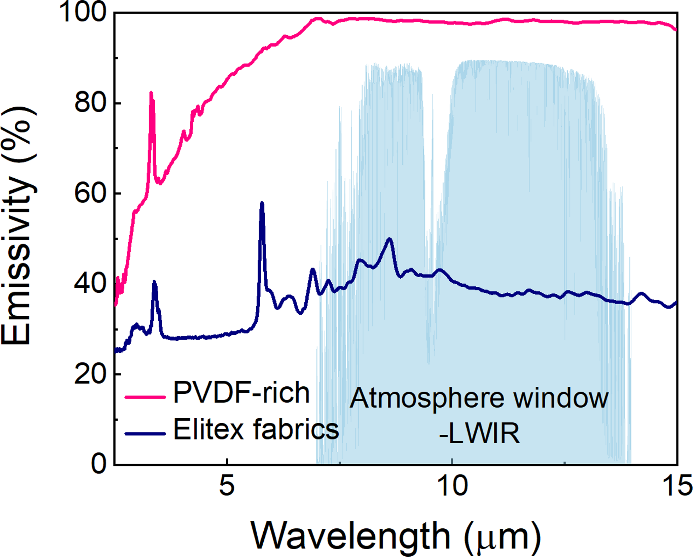


**Fig. S22.** Emissivity for the PVDF-rich side of GMFT and Elitex fabrics.


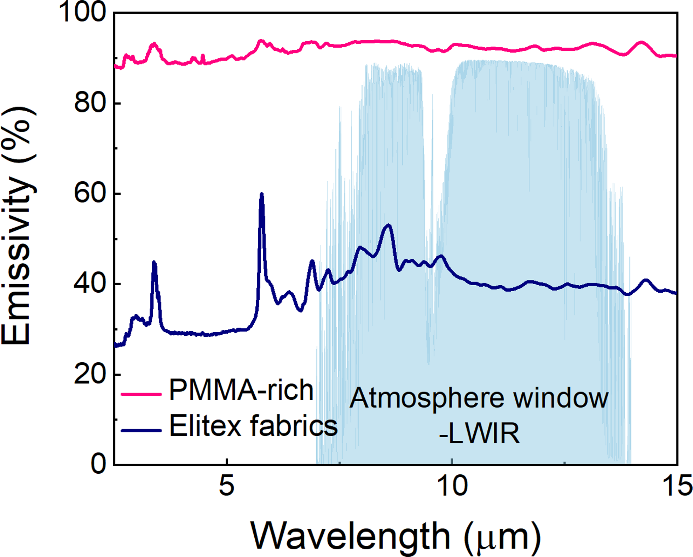


**Fig. S23.** Emissivity for the PMMA-rich side of GMFT and Elitex fabrics.


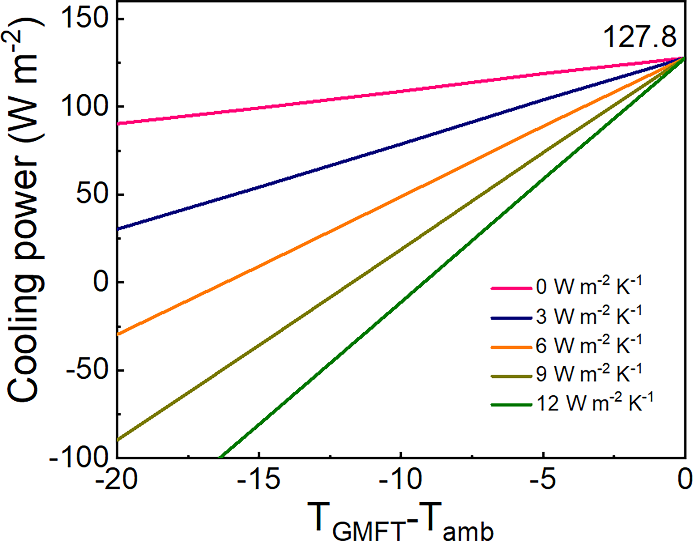


**Fig. S24.** Calculated net nighttime cooling power of the PVDF-rich side of the GMFT.


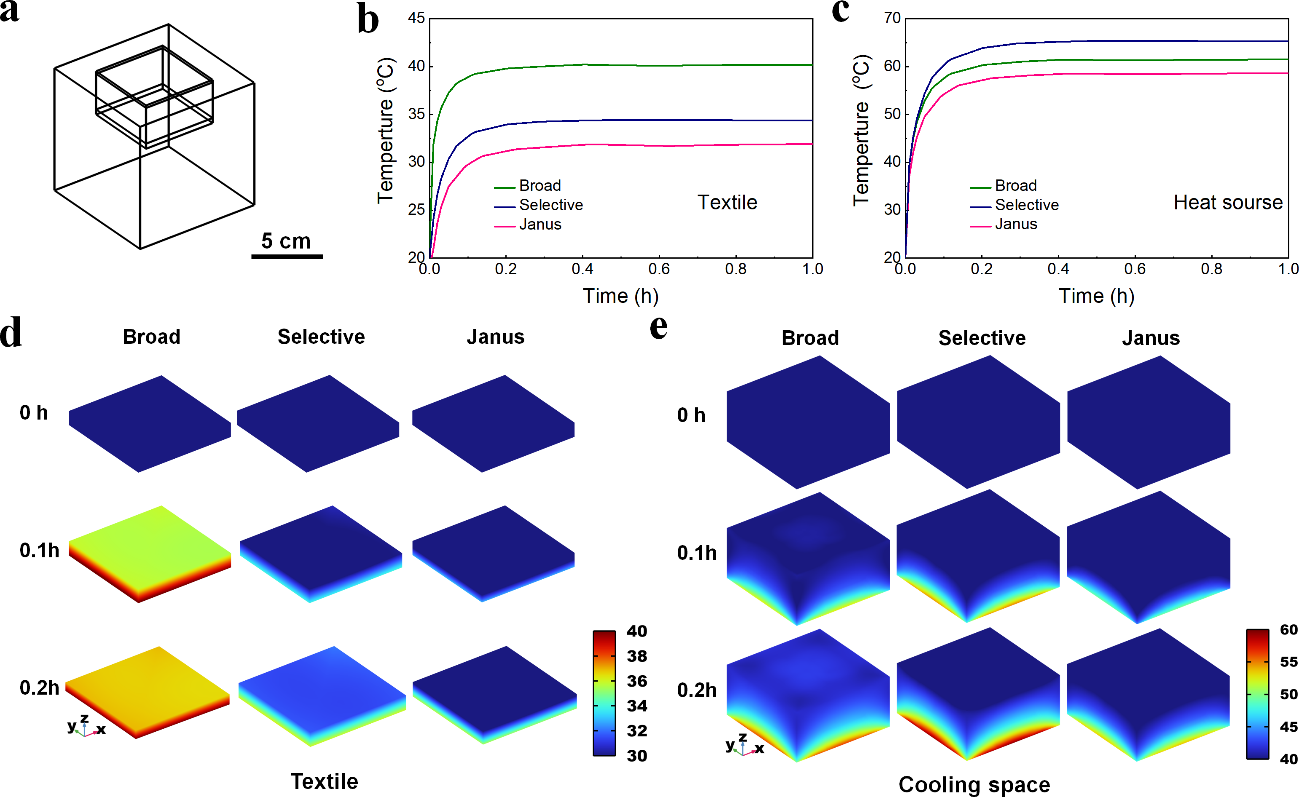


**Fig. S25.** a) Three-dimensional model for the COMSOL simulation of thermal diffusion. b) Simulated temperature profiles of thermal diffusion for textiles with three spectral characteristics (selective, broad, and Janus). c) Temperatures corresponding to a constant power heat source. d) Finite element analysis of the thermal diffusion trends of the three spectral textiles. e) Temperature field of heat diffusion in the cooling space of fabrics with the three spectral properties.


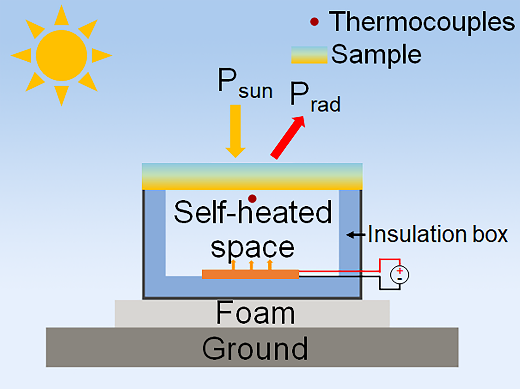


**Fig. S26.** Schematic representation of the experimental setup used to evaluate the above-ambient radiative cooling performance of the GMFT for a self-heated environment.


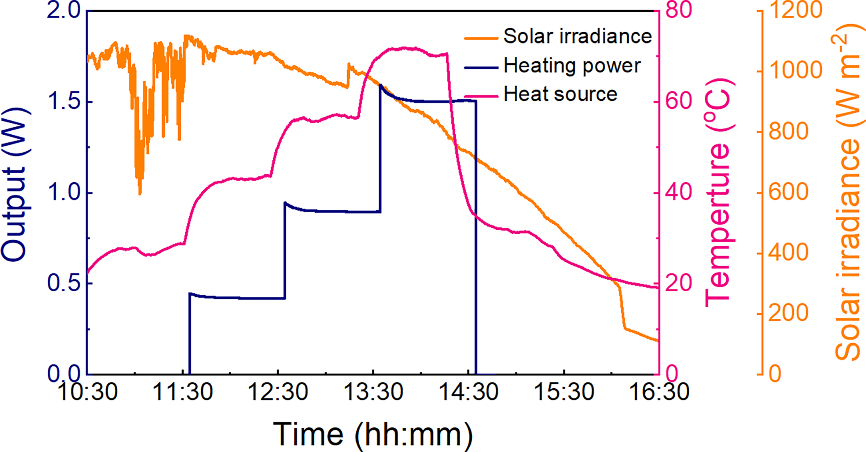


**Fig. S27.** The solar irradiance intensity, the heating power and the temperature of the ceramic heating pad over time profiles.


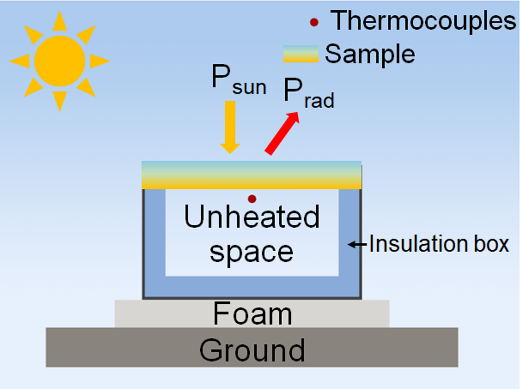


**Fig. S28.** Schematic representation of the experimental setup used to evaluate the sub-ambient radiative cooling performance of the GMFT for an unheated environment.


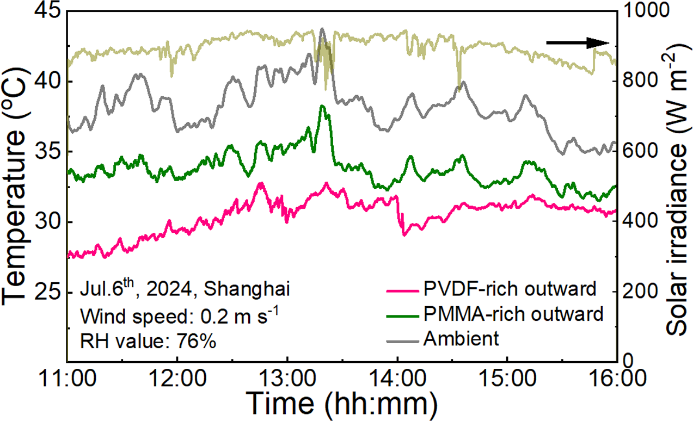


**Fig. S29.** Solar radiation intensity and real-time temperatures of the GMFT during radiative cooling of an unheated space on July 6^th^, 2024 in Shanghai (China).


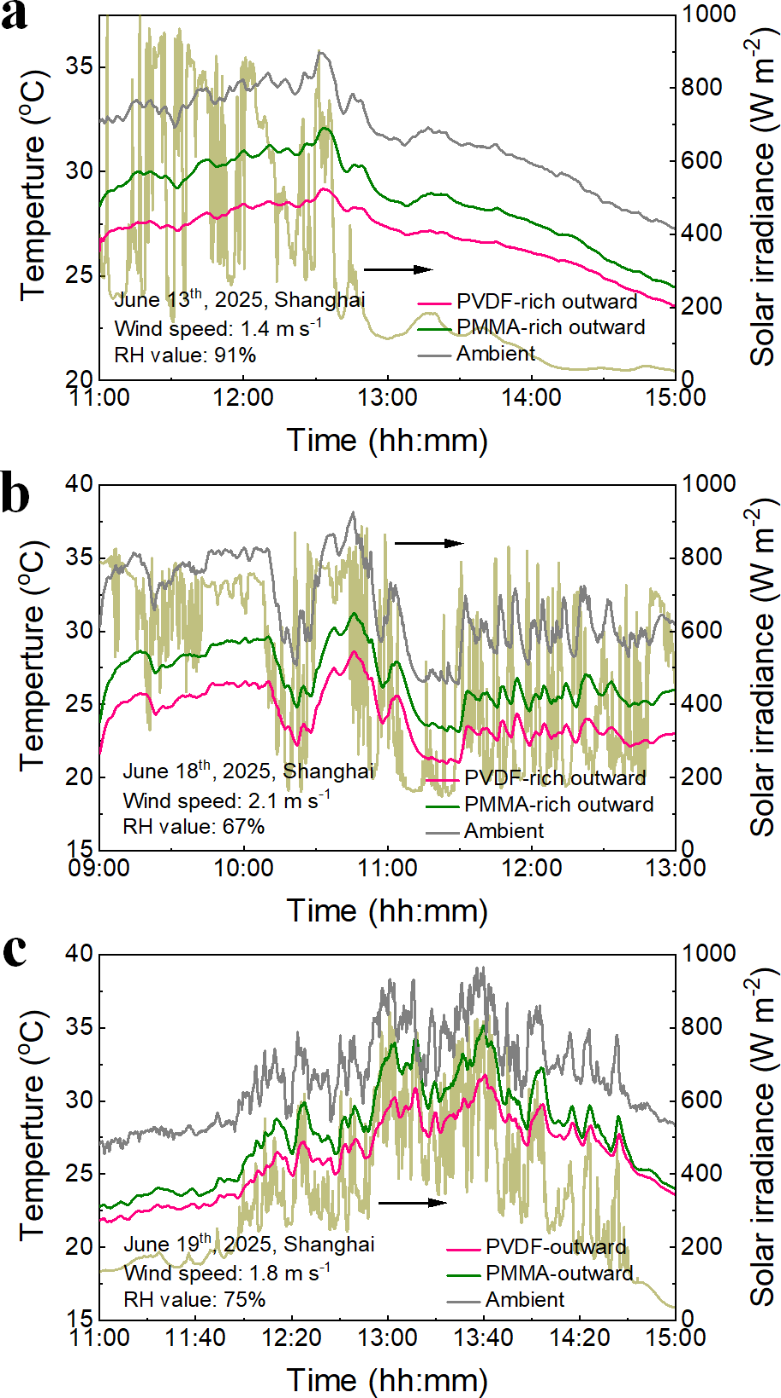


**Fig. S30.** Solar radiation intensity and real-time temperatures of the GMFT during its radiative cooling of unheated spaces on a) June 13^th^, b) June 18^th^, and c) June 19^th^ in 2025, in Shanghai (China).


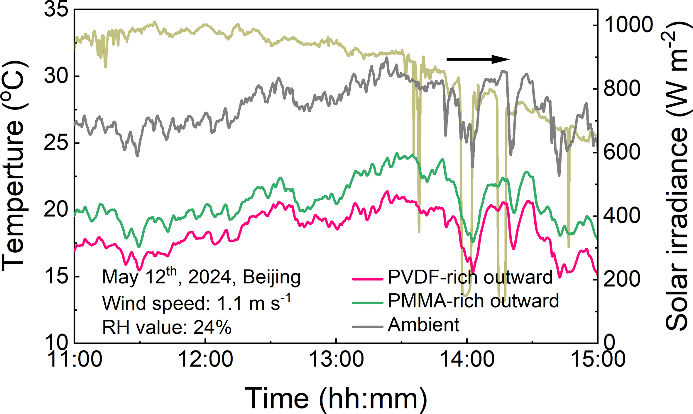


**Fig. S31.** Solar radiation intensity and real-time temperatures of the GMFT during its radiative cooling of an unheated space on May 12^th^, 2024 in Beijing (China).


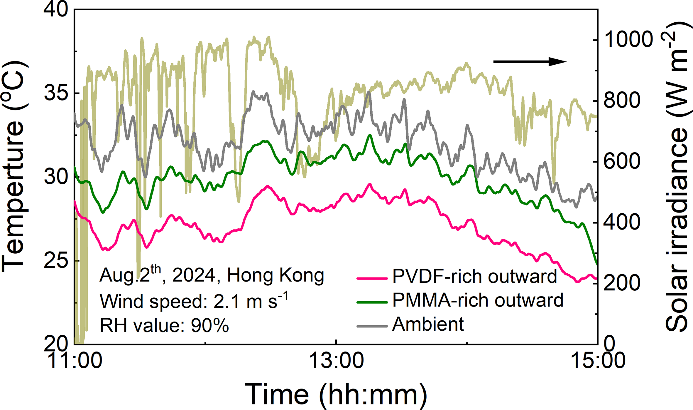


**Fig. S32.** Solar radiation intensity and real-time temperatures of the GMFT during its radiative cooling of an unheated space on August 8^th^, 2024 in Hong Kong (China).


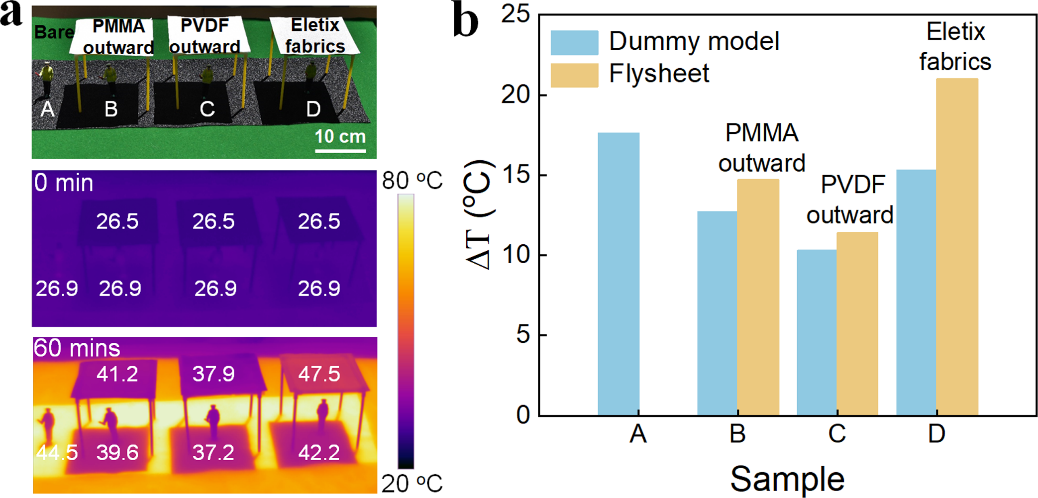


**Fig. S33.** a) Infrared images illustrating the thermal profiles of a bare dummy model and three additional dummy models covered with PMMA-rich, PVDF-rich, and Eletic fabrics flysheets, before and after 60 minutes of exposure to direct sunlight. b) Differences in surface temperature between the dummy models and the flysheets before and after 60 minutes of direct sunlight.


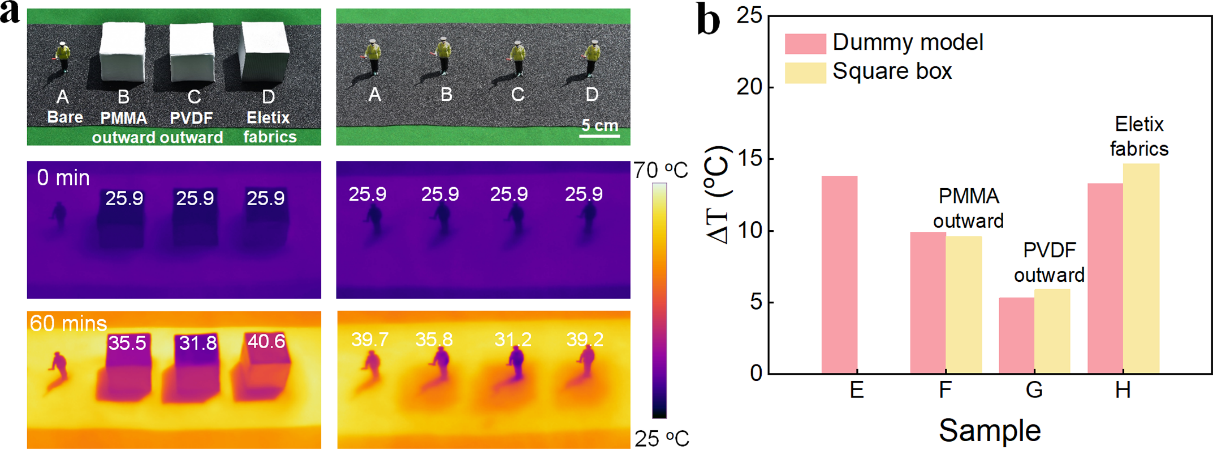


**Fig. S34.** a) Infrared images illustrating the thermal profiles of a bare dummy model and three additional dummy models covered with PMMA-rich, PVDF-rich, and eletic fabric boxes, before and after 60 minutes of exposure to direct sunlight. b) Differences in surface temperature between the dummy models and the square boxes before and after 60 minutes of direct sunlight.


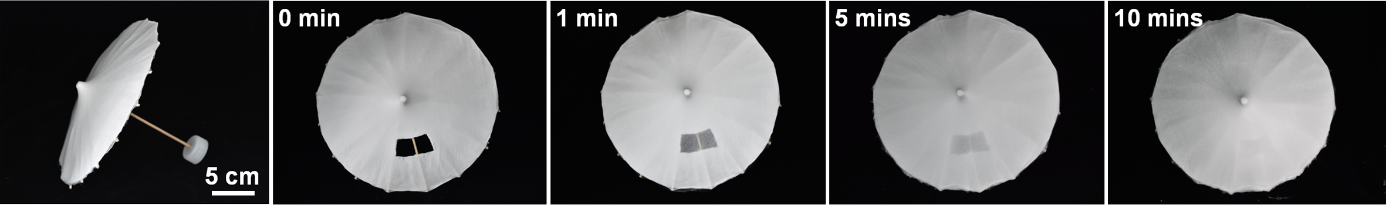


**Fig. S35.** Photographs of the GMFT umbrella model with broken box shapes undergoing the in-situ blow-spun healing process.


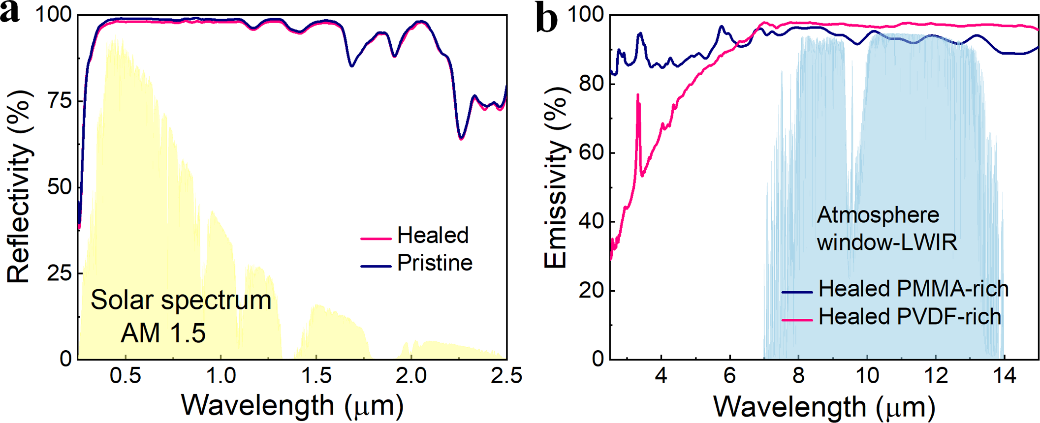


**Fig. S36.** a) UV-vis-NIR reflectance for pristine and healed GMFT. b) Emissivity for the PMMA-rich and PVDF-rich sides in the healed GMFT.


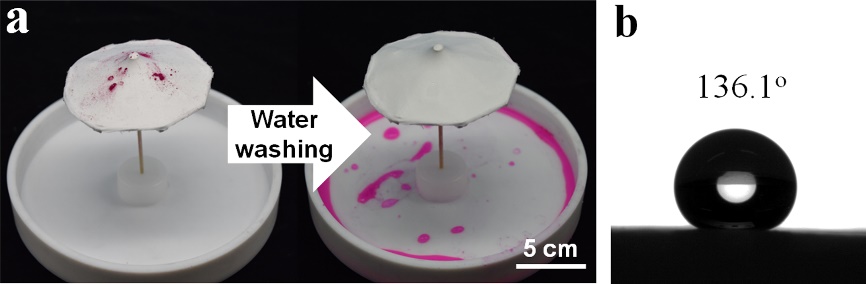


**Fig. S37.** a) Photograph demonstrating the self-cleaning performance of the healed GMFT umbrella model. b) Water contact angle of the PVDF-rich side of the healed GMFT.


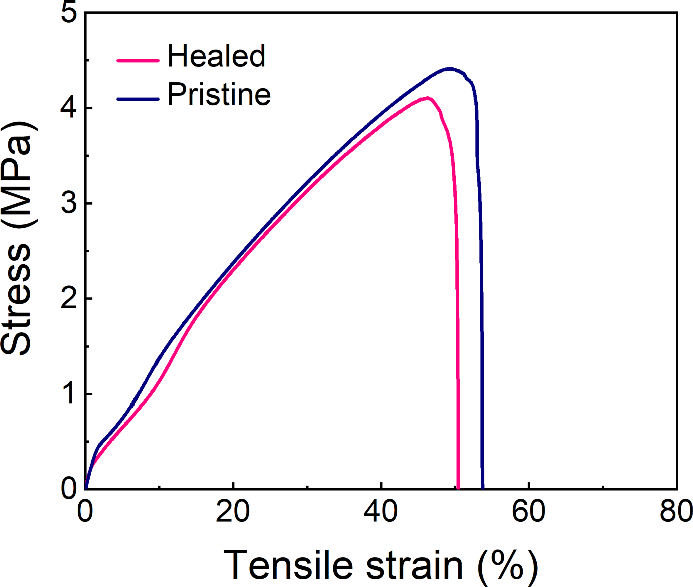


**Fig. S38.** Stress-strain curves of the pristine and healed GMFT.


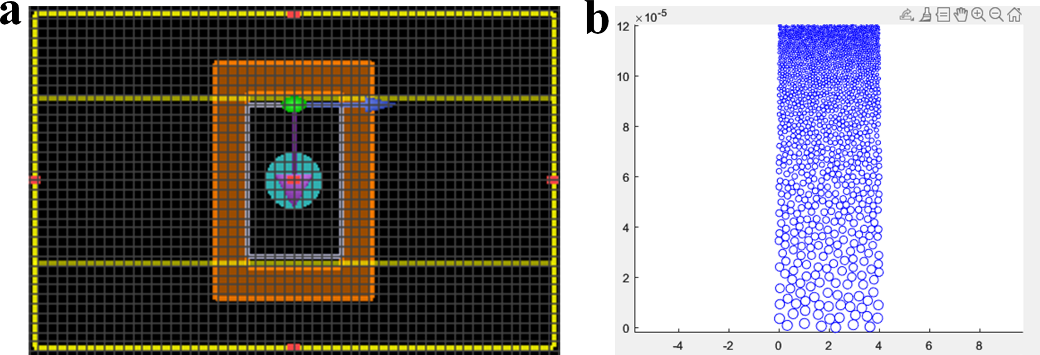


**Fig. S39.** a) Three-dimensional and b) two-dimensional models for the FDTD.


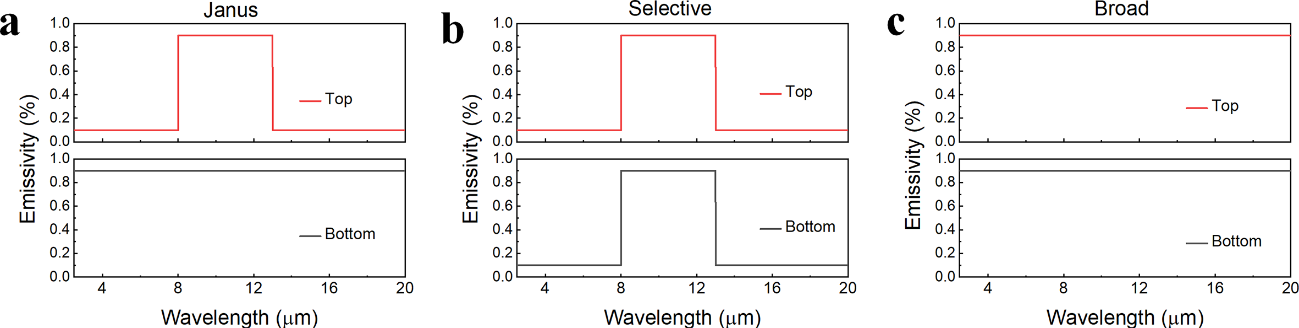


**Fig. S40.** COMSOL calculated emissivity of various spectral textiles: a) Janus, b) Selective, c) Broad.
